# Fuzzy-clustering–based multi-scale intrusion detection for the industrial internet of things

**DOI:** 10.1371/journal.pone.0362087

**Published:** 2026-11-06

**Authors:** Yuhan Yang, Damin Zhang, Qing He, Mu Lin, Ji Zhao

**Affiliations:** College of Big Data and Information Engineering, Guizhou University, Guiyang, China; University of the West of Scotland, UNITED KINGDOM OF GREAT BRITAIN AND NORTHERN IRELAND

## Abstract

The Industrial Internet of Things (IIoT) faces severe intrusion-detection challenges under multi-protocol, multi-device, and massive heterogeneous traffic, especially extreme class imbalance and poor minority-class detection. To address this, we propose a fuzzy-clustering-based multi-scale intrusion detection model (FPBW-IDS). At the data level, we introduce boundary-prioritized FCM-SMOTE: fuzzy memberships distinguish core vs. border samples, interpolation is prioritized in high-membership regions, and sampling weights are adjusted by membership, thereby significantly enhancing minority-class representation and suppressing noise diffusion while keeping the majority class fixed at a multiplier of 1.0. At the feature-learning level, we build three parallel branches across the spatial, temporal, and frequency domains: parallel Pyramid Depthwise Separable Convolutions (PDSC) to capture spatial (cross-feature) dependencies, BiGRU to model temporal dependencies, and wavelet transforms to extract multi-scale spectral statistics; their outputs are fused with learned weighting to form a complementary tri-domain representation. Experiments on the CICIDS2017 dataset show that the proposed method achieves state-of-the-art performance under both unsampled and sampled settings: Accuracy 99.39%, Precision 98.22%, Recall 93.69%, F1 95.56% in the comprehensive comparison; and Accuracy 98.97%, Recall 99.09%, F1 96.69% in a recall-oriented setting, markedly reducing missed detections while maintaining stable training/validation curves and a small generalization gap. These results show the effectiveness and reliability of clustering-guided oversampling combined with tri-domain multi-scale representation for IIoT intrusion detection.

## 1 Introduction

The concept of the Internet of Things (IoT) was first proposed by Ashton in 1999 [1] and was initially viewed as a platform enabling physical devices to connect with each other over the Internet [2]. With the rapid development of Internet and sensing technologies, IoT has gained a lot of attention. Thanks to stronger 5G and expanding internet services, IoT is scaling up, connecting things more tightly, and becoming more important in industrial change [3–5].

The Industrial Internet of Things (IIoT) refers to the close interconnection of sensors, devices, machines, and information technology systems through the Internet, creating an intelligent industrial ecosystem. IIoT integrates data capture, live monitoring, practical analysis, and decision support. It improves the whole production flow, supports automation, and raises efficiency and innovation. The *Notice on Issuing the Development Plan for the Information and Communication Industry (2016–2020)* by the Ministry of Industry and Information Technology emphasizes the critical value of industrial Internet security. In the current field of cybersecurity, exploring the use of emerging technologies such as artificial intelligence to improve security protection is particularly important [6–8]. Industrial Internet security plays a vital role in building a strong manufacturing nation and a strong cyber nation, serving as a key support for achieving high-quality economic development in China. With the transformation of industrial manufacturing toward intelligence and digitalization, not only has production scale expanded and efficiency improved, but industrial competitiveness has also been greatly enhanced. However, since IIoT combines physical devices, network communication, and digital control [9], it forms a highly complex and interconnected system. This multi-layered architecture increases system complexity and exposes the Industrial Internet of Things to security threats.

However, as the volume of data stored by the Industrial Internet of Things (IIoT) increases, its risk of suffering cyberattacks rises significantly, posing substantial threats to enterprise management and production. Meanwhile, with the continued expansion of the IIoT and the participation of an ever-growing number of industries, its network architecture has become increasingly complex, thereby raising the requirements for defensive capabilities [10–13]. The combined impact of these factors has made IIoT security increasingly urgent, and research on related intrusion detection technologies has consequently become a current hotspot.

An Intrusion Detection System (IDS) is a software or device used to monitor and defend against network intrusions and malicious activities. It identifies anomalies by analyzing network traffic or system logs, and once suspicious activity is detected, it sends an alert to the management unit. IDS mainly adopts two detection methods: signature matching and anomaly detection. Signature matching (or rule-based matching) IDS identifies known attacks by comparing against a pre-stored database of attack signatures [14], and can maintain a relatively high detection rate even on resource-constrained devices [15]. However, since it relies entirely on existing signatures, this method is powerless against zero-day or unrecorded attacks, and it requires continuous updates and maintenance to preserve its protective effectiveness.

Current intrusion detection techniques include approaches based on machine learning and deep learning; here we introduce only machine learning and deep learning:

(1) Machine Learning: Mukherjee S, Sharma N [16] used the Naive Bayes classifier in Weka 3.6 and conducted 10-fold cross-validation on the NSL-KDD dataset. They compared the full feature set with four dimensionality reduction methods: CFS, Information Gain, Gain Ratio, and the authors’ proposed “Feature Vitality Based Removal Method” (FVBRM). For each step of removing a single feature, they performed feedback evaluation on model performance (accuracy, RMSE, average true positive rate). Ultimately, with only 24 features retained, the FVBRM + Naive Bayes model achieved an overall accuracy of 97.78%, significantly outperforming other methods (e.g., 95.11% with the full feature set); Zou, Luo et al.[17] innovatively combined bottom-up hierarchical clustering with a decision tree structure. By calculating inter-cluster similarity, they dynamically merged categories to form a multi-class decision tree, embedding Twin Support Vector Machines (TWSVM) at each non-leaf node. This design balanced noise robustness with computational efficiency, requiring only *K–1* binary sub-models to achieve efficient and fine-grained multi-class network intrusion detection; Jiang, He et al.[18] proposed a PSO-XGBoost hybrid model, where Particle Swarm Optimization (PSO) adaptively searched for key hyperparameters of XGBoost (learning rate, tree depth, leaf weight, gamma, subsample ratio, and column sampling ratio). This approach improved both overall classification performance and the detection of minority classes (such as U2R and R2L).
(2) Deep Learning: Chen, Lv et al.[19] proposed an anomaly network traffic detection model based on a Bidirectional Temporal Convolutional Network (BiTCN). By combining bidirectional architecture with dilated convolution, the model captured both forward and backward semantic features of traffic, significantly enhancing detection accuracy and robustness; Shu, Zhou et al.[20] proposed a collaborative intrusion detection system (CIDS) based on distributed SDN and deep learning. By incorporating a Multi-Resolution Generative Adversarial Network (MD-GAN), multiple distributed SDN controllers collaboratively trained a global intrusion detection model, avoiding traffic bias issues found in traditional methods. This system reduced computation and communication overhead through distributed learning and parameter exchange, while ensuring efficient detection performance; Xie, Wang et al.[21] proposed a multivariate anomaly detection method that combined 1D-CNN and GRU. Using an autoencoder for feature learning and dimensionality reduction, and applying statistical deviation for anomaly detection. The results showed that this method worked much better than traditional approaches on the SWaT dataset, especially in finding new and unknown attacks.

Despite the considerable progress achieved in network intrusion‑detection research, several practical challenges still remain to be addressed for real‑world traffic‑analysis scenarios. First, real‑world intrusion datasets such as CICIDS2017 exhibit severe class‑imbalance characteristics, where rare attack categories contain far fewer samples compared with normal traffic. Directly adopting vanilla SMOTE for oversampling tends to generate noisy synthetic samples near class boundaries and distorts the intrinsic data manifold, which degrades classification performance for minor‑class attack instances. Second, most existing hybrid temporal models, such as CNN‑LSTM architectures, mainly extract spatial and temporal features from network‑traffic sequences, while ignoring frequency‑domain statistical patterns hidden within time‑series traffic data. The multi‑scale fluctuation and burst characteristics reflected in spectral information cannot be sufficiently captured, resulting in incomplete feature representation. Third, many existing studies either focus only on oversampling optimization or solely improve network backbone modules, and rarely jointly optimize the imbalance‑processing strategy and multi‑domain feature‑extraction branches for network‑intrusion detection. To remedy the above drawbacks, this paper addresses the following core research issues: how to construct an effective oversampling strategy that distinguishes core interior samples and boundary samples of minority classes, and how to design a multi‑branch network to jointly exploit spatial, temporal, and frequency‑domain information from network‑traffic sequences for improving multi‑class intrusion‑detection performance under imbalanced data distribution.

At present, various network intrusion detection systems based on machine learning and deep learning have been developed. Although there have been important achievements, many key issues are still unresolved. Problems such as overfitting caused by redundant data features and class imbalance—particularly the low detection rate of minority attack classes—limit the accuracy of models. Therefore, effectively addressing the issue of data imbalance and improving the detection rate of minority attack classes have become key challenges in enhancing the performance of network intrusion detection systems. Data resampling is a widely used method for handling imbalanced data. The main idea is to adjust the sample distribution through resampling techniques so that different classes of data reach a relatively balanced state. Among these, the SMOTE (Synthetic Minority Over-sampling Technique) algorithm is a particularly important method. Many researchers have employed SMOTE and its improved variants to address the data imbalance problem [22,23]. The traditional SMOTE method generates new samples by performing linear interpolation between minority-class samples and their *k* nearest neighbors. If the *k* neighbors are highly similar to one another, the synthesized samples may lack diversity and fail to effectively reflect the true distribution of the minority class, thereby undermining the model’s generalization ability.

In traditional intrusion detection techniques, feature extraction that combines temporal and spatial features is the most widely used, followed by combinations of temporal and frequency features. Han, Cheng et al. [24] employed an attention-based convolutional network and an attention-enhanced LSTM to simultaneously analyze spatial and temporal features. The attention mechanism enabled the system to focus on key bytes in CAN messages, reducing the impact of irrelevant data and thereby improving detection accuracy. Huang, Zhao et al. [25] proposed Fused-ConvLSTM, which integrates depthwise separable convolution, bottleneck structures, and SE attention. This model efficiently propagates spatiotemporal information across sequential frames while refining features, striking a balance between detection accuracy and computational efficiency.

Network traffic sequences naturally contain multi‑scale statistical patterns. Bursty attack behaviours produce short‑term high‑frequency statistical fluctuations, whereas normal network activities maintain relatively stable long‑term low‑frequency statistical baselines. Therefore, traffic samples contain distinguishable information across temporal, spatial and statistical‑frequency dimensions. Nevertheless, most existing intrusion‑detection models only exploit spatial and temporal representations while neglecting frequency‑domain statistical patterns, failing to fully mine complementary multi‑scale information hidden within traffic data. To address the above challenges, the contributions of this paper are as follows:

(1) This paper first uses Fuzzy C-Means (FCM) to compute the membership of minority-class samples and distinguish central from boundary samples. It then focuses on interpolating in areas with high membership and adjusts the sampling weights based on membership, to keep the minority class structure and reduce noisy overlap.
(2) This paper innovatively extracts features separately from the temporal, spatial, and frequency domains. These three types of features are then either combined at the sample level or processed in parallel via a multi-branch network, followed by weighted aggregation through a fusion layer or an attention mechanism. In this way, at the same time the model learns across all three dimensions, thereby substantially improving intrusion detection accuracy and localization precision.
(3) This paper introduces the wavelet transform to extract frequency‑domain statistical features. Leveraging multi‑scale time‑frequency statistical analysis, the wavelet transform can precisely capture transient statistical fluctuations of attack traffic across multiple scales, suppress noise interference, and generate rich statistical descriptors, so as to improve detection performance by exploiting frequency‑related statistical patterns hidden in reordered traffic time‑series samples.

## 2 Related work

### (1) Processing imbalanced datasets

Aiming at the class‑imbalance issue in network intrusion detection, Maseer et al. [26] performed benchmark evaluations of 31 configurations of traditional machine‑learning and CNN models on the multi‑class CIC‑IDS2017 dataset without imbalance‑optimization strategies. Their work reveals drastic sample‑size discrepancies across classes, where rare attacks such as Bot and Web Attack account for very small proportions. Overall accuracy cannot serve as a reliable metric: most models perform well on majority‑class traffic yet suffer obvious drops in recall and F1‑score for minority attacks. Even deep‑learning models may produce majority‑biased decisions without handling sample‑distribution shift, raising the risk of missed small‑sample attacks. This benchmark indicates that network‑architecture optimization alone cannot resolve imbalanced detection, and resampling strategies at the data level are essential to mitigate performance degradation caused by uneven sample distribution.

To mitigate class imbalance, many studies combine vanilla SMOTE oversampling with temporal deep‑learning models for network intrusion detection. Singh et al.[27] adopted a hybrid CNN‑LSTM temporal network and employed SMOTE to generate synthetic minority samples, and validated the effectiveness of this combined scheme on the multi‑class CICIDS2017 dataset. Nevertheless, vanilla SMOTE applies uniform nearest‑neighbor interpolation for all minority instances without considering multi‑cluster patterns within minority classes. It tends to produce noisy synthetic samples near class boundaries and may distort the original data manifold, which degrades the learning of classification boundaries for subsequent deep‑learning models. Furthermore, such hybrid architectures mostly focus on spatial and temporal representations of traffic sequences while ignoring frequency‑domain statistical information, and thus cannot fully exploit multi‑scale fluctuation characteristics hidden in network traffic.

Zhang, Huang et al.[28] proposed a new class-imbalance handling method, SGM. First, SMOTE oversampling is applied to the minority-class samples to generate synthetic instances; then the majority-class samples are clustered using a Gaussian Mixture Model (GMM), followed by uniform downsampling within each cluster. This approach avoids the excessive redundancy introduced by pure oversampling and mitigates the loss of critical information that can result from random downsampling, thereby significantly improving the detection rate of minority classes.Yan, Han et al.[29] improved upon the traditional SMOTE oversampling method’s “one-size-fits-all” global strategy and proposed the RA-SMOTE algorithm. RA-SMOTE analyzes the neighborhood distribution of each minority-class sample and categorizes them into three regions: isolated points, safe points, and risky points. It then applies differentiated synthesis strategies accordingly—interpolation along connecting lines in isolated regions containing only minority samples, directed generation based on cluster centers in risky regions with boundary overlap, and no oversampling in safe regions. This region-adaptive approach not only preserves the multimodal distribution structure of the minority class but also effectively suppresses the erroneous amplification of boundary and noisy samples. Combined with feature selection, it further enhances the efficiency and accuracy of intrusion detection. Xu, Zhao et al.[30] proposed an imbalanced data processing method called CInfFS-SPISE. The method first applies random sparse projection on high-dimensional traffic features combined with cluster-based Inf-FS feature selection to eliminate redundancy and enhance representational capability. It then employs the SPISE algorithm to dynamically select the most representative subset under dual criteria of diversity and similarity, thereby achieving fine-grained data balancing. Finally, both components are jointly embedded into the intrusion detection model training process. This approach not only ensures diversity and discriminative power at the feature level but also significantly improves detection accuracy (G-Mean ≥ 99.62%) and robustness (FAR < 0.7%) through dynamic subset construction.

Several studies have integrated clustering strategies with SMOTE to mitigate class‑imbalance issues. Pruengkarn et al.[31] proposed FCMSMT, a hybrid under‑over‑sampling scheme based on fuzzy c‑means and SMOTE for multiclass imbalance tasks. It adopts the average sample count across all classes as the balancing target: FCM‑driven undersampling is applied for over‑represented majority classes while SMOTE oversampling is executed for minority classes. Nevertheless, FCMSMT treats all minority instances equally and does not leverage per‑sample fuzzy‑membership information to differentiate core and boundary samples, which may still yield noisy synthetic samples in heavily overlapping regions. As a representative hard‑partition clustering variant, k‑means‑SMOTE proposed by Douzas et al.[32] filters unsafe clusters dominated by majority‑class instances and only performs SMOTE oversampling within minority‑dominated safe clusters, assigning more synthetic instances to sparse minority sub‑clusters to ease intra‑class imbalance. However, hard k‑means cannot quantify the fuzzy belonging degree of borderline samples; it assigns identical weights to all samples inside a qualified cluster without fine‑grained sample‑level evaluation, leaving risks of generating low‑quality synthetic samples under severe class overlap. Different from the above two schemes, our proposed method calculates fuzzy‑membership value for each minority‑class instance and assigns adaptive oversampling weights accordingly, giving higher interpolation priority to high‑membership core samples and suppressing noisy interpolation near ambiguous boundaries.

### (2) Anomaly detection

In recent years, the detection of abnormal electricity consumption behaviors has attracted widespread attention. Zhang, Dong et al.[33] proposed an unsupervised feature‑engineering‑based framework for detecting anomalous electrical behavior. They argue that traditional studies focus solely on improving model architectures while neglecting advances in feature engineering. In their approach, an initial large‑scale raw feature set is automatically generated from time‑series load profiles using the tsfresh toolkit. Then, leveraging nonlinear correlations among features measured by the maximal information coefficient, an optimal feature set that reflects users’ electricity consumption behavior is selected under an unsupervised paradigm. Finally, at the prediction stage, a density‑based clustering algorithm is used to detect anomalous electricity usage. Nevertheless, the method was only validated on small‑scale industrial‑park datasets, and the clustering component may suffer computational bottlenecks when handling massive‑volume time‑series data.

In addition to fully‑supervised temporal detection models, some works explore semi‑supervised temporal intrusion detection to cope with label scarcity, adversarial contamination and temporal distribution drift. Chattopadhyay et al.[34] proposed RSST‑NIDS, a robust semi‑supervised temporal framework combining confidence‑aware pseudo‑labeling, consistency regularization and selective temporal invariance. It filters adversarially‑polluted unlabeled samples via multi‑criteria screening to improve robustness in cloud‑network scenarios. Nevertheless, this framework targets binary classification (benign vs malicious traffic). As pointed out in their original paper, pseudo‑label confidence for minority attack classes cannot be guaranteed when extended to multi‑class imbalanced scenarios, which limits its applicability for fine‑grained seven‑attack‑type recognition. Accordingly, this paper adopts a fully‑supervised paradigm and constructs a three‑branch parallel fusion network covering spatial‑temporal‑frequency domains to realize fine‑grained multi‑class classification for IIoT traffic.

Traffic anomaly detection for network security has always been a critical research topic, and numerous spatiotemporal‑based intrusion detection approaches have been developed in recent years. Zhong et al.[35] innovatively constructed a hybrid MSCNN‑MLFLSTM network for network traffic anomaly detection. Previous spatiotemporal IDS approaches generally built feature extraction pipelines based on ordinary single‑scale convolution and vanilla stacked LSTM, which fail to capture multi‑granular spatial patterns and suffer from limited capability for imbalanced minority‑attack identification. In contrast, they adopted multi‑scale 1‑D convolution to extract multi‑granularity spatial representations and stacked multi‑layer‑fusion LSTM to model temporal dependencies, further combining BYOL pre‑training and Focal Loss to boost overall detection performance. Nevertheless, it ignores frequency‑domain information and handles class imbalance merely via loss‑level weighting instead of data‑driven resampling.

Public safety and the monitoring of anomalous events in crowded places have always been crucial, and in recent years many crowd anomaly detection methods have been proposed. Wei et al. [36] innovatively introduced a pretrained two-stream fully convolutional network (FCN) into crowd anomaly detection. Previous approaches typically engineered complex hand-crafted features for surveillance videos, but these features were affected by computational complexity and scene variations, leading to suboptimal detection accuracy. In contrast, they used a two-stream fully convolutional neural network to directly extract appearance and motion features from video frames and optical flow, thereby significantly enhancing anomaly detection performance.

Khan S W, Hafeez Q et al. [37] explored deep‑learning‑driven anomaly detection for traffic surveillance video scenarios. They adopted a ResNet50 transfer‑learning backbone for frame‑level anomaly classification and designed a rolling‑prediction‑averaging strategy to mitigate label‑flickering artifacts caused by independent per‑frame predictions. Nevertheless, their solution targets visual video data rather than network traffic sequences. The visual features extracted from surveillance frames cannot be directly migrated to packet‑level network intrusion detection, and the model still suffers obvious performance degradation under low‑quality, long‑distance and complex environmental conditions, which limits its direct applicability to cybersecurity traffic‑analysis tasks.

### (3) Abnormal network traffic detection in the industrial Internet of Things (IIoT)

Telikani et al. [38] addressed the data imbalance problem in the Industrial Internet of Things (IIoT) by proposing a solution for selecting the optimal features from the dataset. The study constructed a hybrid model that combines a stacked autoencoder with a CNN, and optimized its parameters using a cost-sensitive loss function. Extensive experiments show that this approach can significantly alleviate the imbalance of IIoT traffic data while offering excellent expandability.

Zhang et al. [39] presented an intrusion detection scheme suited for small-scale IIoT devices: each device locally fine-tunes its model using a transfer learning approach, then sends the model information to a central server. The server aggregates them—voting based on individual model performance—to form a stronger model. The authors validated this method on a Raspberry Pi testbed and with real traffic data, showing that it not only protects device privacy but also substantially improves intrusion detection accuracy and stability.

To alleviate the tension between dimensionality reduction and feature preservation in imbalanced industrial big data, Zhou et al. [40] proposed a Variational LSTM (VLSTM) anomaly detection model based on reconstructed feature representations. They designed an encoder–decoder neural network incorporating a variational reparameterization scheme to learn low-dimensional feature representations from high-dimensional raw data. Meanwhile, they defined and quantified three loss functions to constrain the reconstructed latent variables, making them more interpretable and discriminative. Finally, the optimized low-dimensional features are fed into a lightweight discriminative network to efficiently identify anomalous behaviors in industrial big data.

Gao et al. [41] constructed a dataset of 8,177 images collected from 653 cameras, focusing on the challenges of intrusion detection in power grid monitoring scenarios, where target scales are diverse and application environments are complex. On this basis, they proposed ID-Net, an improved Mask R-CNN model: it introduces modulated deformable convolution in the backbone network to adapt to target deformations, employs a self-attention module to model long-range contextual dependencies, achieves multi-scale fusion through a feature pyramid, and refines bounding box regression step by step using a cascaded RPN. Experimental results demonstrate that the model achieves competitive performance.

Yang et al. [42] designed a novel intrusion detection framework. First, they integrated an attention mechanism, bidirectional gated recurrent units (BiGRU), and an Inception-CNN structure to enhance the capability of identifying anomalous traffic. Next, hybrid sampling and denoising were applied to mitigate the bias caused by data imbalance and to eliminate sampling noise. Finally, Pearson correlation analysis combined with random forest was used to select key features and remove redundant information, enabling the model to more accurately extract essential elements from high-dimensional attack traffic.

Most of the above anomaly detection models rely on temporal and spatial features while neglecting frequency-domain features, thereby losing the interactions among features. In addition, intrusion detection models that target only a single scale cannot cope with changes in the operating environment and architecture of the IIoT; once new attack methods emerge, the models must be continually adjusted, and may even fail to detect such attacks. Moreover, because only a very limited number of anomalous traffic samples are available during training, the models struggle to accurately capture their underlying distributions, resulting in poor detection performance when samples are scarce.

## 3 Methods

In this section, we first describe how to obtain traffic data from the IIoT platform. Then, we present the implementation details of the FPBW-IDS model, including how to perform multi-scale feature extraction and how to use a fuzzy clustering algorithm to increase the data volume of anomalous minority-class samples.

### (1) Capturing data with IIoT

The Industrial Internet of Things comprises a three-layer architecture: the bottom layer is the device layer, covering various sensors and terminals; the middle layer is the gateway layer, which performs data parsing and forwarding via wireless access, gateways, and switching devices; and the top layer is the cloud service layer, which combines and stores all traffic information and runs a variety of algorithms on this basis to support business functions. We select traffic data from this top layer as the training set for the FPBW-IDS model. In addition, introducing blockchain technology can further enhance the platform’s data security capabilities.

### (2) Minority-class synthetic sampling algorithm optimized via fuzzy clustering

Traditional SMOTE often suffers from problems such as noise strengthening, fuzzy class boundaries, and distorted data distributions. To address these shortcomings, researchers have embedded clustering mechanisms [43,44] into the oversampling process: clustering is first used to delineate a “clean region,” and then SMOTE is applied within that region, thereby reducing the side effects caused by randomness at the source. It should be noted, however, that while clustering improves sampling effectiveness, it also introduces new issues—K-means is highly sensitive to the selection of initial centroids.

K-means is a prototype-based unsupervised clustering method, aiming to section data into K clusters such that samples within the same cluster are as similar as possible, while differences between clusters are maximized. It is commonly used for fast clustering of large-scale datasets. K-means must rely on initial centroids to determine dividing, which are then iteratively optimized. If the initial centroids are chosen well, K-means can quickly converge to a near-optimal clustering result; however, if chosen poorly, the algorithm may fall into a “local optimum,” leading to poor clustering performance. Moreover, since the algorithm must repeatedly adjust and update centroids on large-scale data, it often incurs a high computational cost.

K-means is a typical hard clustering algorithm, which assigns each traffic sample to only one cluster. However, in imbalanced intrusion detection datasets such as CICIDS2017, the feature distributions of minority attack samples often overlap with normal traffic. The hard partition mechanism of K-means is prone to misclassify boundary ambiguous samples, which will degrade the quality of synthetic samples generated in the subsequent SMOTE oversampling stage. In contrast, FCM is a soft clustering method that calculates the membership degree of each sample to all clusters. It can better characterize the fuzzy boundary between normal flows and rare attack flows, so as to generate more reliable minority samples when combined with SMOTE. Therefore, FCM is adopted as the clustering component in our oversampling framework.

#### ⅰ) Definition of FCM.

Fuzzy C-Means (FCM) clustering is a classic algorithm based on fuzzy set theory. Unlike traditional hard-partitioning methods, FCM represents a sample’s cluster membership by a membership degree: each sample can simultaneously belong to multiple clusters with different degrees, each lying in the range [0,1]，and the memberships across all clusters sum to 1. The algorithm iteratively optimizes a weighted within-cluster sum-of-squares objective, alternating between updating cluster centroids and the membership matrix until convergence. Thanks to this “soft partitioning” mechanism, FCM can better characterize the ambiguity and uncertainty at cluster boundaries, and has therefore been widely applied in pattern recognition, medical image segmentation, and the handling of imbalanced data.

#### ⅱ) Membership-guided sampling weight.

After obtaining the membership matrix U through Fuzzy C-Means clustering, the fuzzy membership information is further incorporated into the oversampling process to adaptively determine the importance of minority-class samples. Unlike traditional SMOTE, which selects minority samples with equal probability, the proposed method assigns different sampling probabilities according to the uncertainty of each sample’s cluster membership.

For the i-th minority-class sample $x_{i}$, the maximum membership degree among all clusters is defined as:


$$\begin{array}{c} m_{i}=\underset{k}{max}\left(u_{ik}\right) \end{array}$$ (1)

where $u_{ik}$ denotes the membership degree of sample $x_{i}$ belonging to the k-th cluster. A larger $m_{i}$ indicates that the sample is located in a more compact cluster region, while a smaller value implies that the sample lies closer to the fuzzy boundary between clusters. Since boundary samples contain more discriminative information for improving classification boundaries, the boundary-aware weight of each sample is calculated as:


$$\begin{array}{c} w_{i}=1-m_{i} \end{array}$$ (2)

where $w_{i}$ represents the degree of boundary uncertainty. Subsequently, a softmax normalization function is introduced to convert the boundary weights into sampling probabilities:


$$\begin{array}{c} p_{i}=\frac{\text{exp}\left(\beta w_{i}\right)}{\sum_{j=1}^{N}\text{exp}\left(\beta w_{j}\right)} \end{array}$$ (3)

where N is the number of minority-class samples and β is a scaling factor controlling the influence of membership uncertainty. According to $p_{i}$, samples with higher boundary uncertainty obtain larger probabilities of being selected as interpolation centers during synthetic sample generation.

Furthermore, the interpolation coefficient between the selected sample $x_{i}$ and its neighbor $x_{j}$ is also adjusted according to their boundary weights:


$$\begin{array}{c} \lambda_{i}=\frac{exp\left(\beta w_{i}\right)}{exp\left(\beta w_{i}\right)+exp\left(\beta w_{j}\right)} \end{array}$$ (4)

The generated synthetic sample is then calculated as:


$$\begin{array}{c} x_{new}=x_{i}+\lambda_{i}(x_{j}-x_{i}) \end{array}$$ (5)

Through the membership-guided weighting strategy, the proposed FCM-SMOTE gives higher priority to informative boundary samples while avoiding excessive replication of noisy isolated samples. Therefore, the generated samples can better preserve the intrinsic distribution of minority classes and improve the discriminative capability of subsequent intrusion detection models.

#### ⅲ) Objective function and variables of FCM.

Given data$\;{\left\{x_{i}\right\}}_{i=\text{1}}^{N}$, number of clusters C, and fuzzifier m>1 (commonly m=2):

Objective Function (Weighted Within-Cluster Sum of Squared Distances)


$$\begin{array}{c} J_{m}\left(𝐔,𝐂\right)=\sum_{i=\text{1}}^{N}\sum_{j=\text{1}}^{C}u_{ij}^{m}{\left\|x_{i}-c_{j}\right\|}^{\text{2}} \end{array}$$ (6)

Where $𝐔={\left[u_{ij}\right]}_{N\times C}$ is the membership matrix, and $𝐂={\left\{c_{j}\right\}}_{j=\text{1}}^{C}$ represents the cluster centers.

Constraints


$$\begin{array}{c} \forall i,\sum_{j=\text{1}}^{C}u_{ij}=\text{1},\text{\textasciitilde{}}\text{0}\leq u_{ij}\leq \text{1}. \end{array}$$ (7)

Distance Metric: Euclidean distance by default‖·‖.

To avoid manual pre‑specification of the cluster count for FCM, an automatic hyper‑parameter selection strategy based on silhouette score is applied individually to each minority‑class sample set $X_{\text{min}}\in {\mathbb{R}}^{N\times d}$. Candidate cluster numbers are traversed within the range k∈[2,8].For each candidate k, FCM clustering is carried out to obtain the fuzzy membership matrix $𝐔\in {\mathbb{R}}^{N\times k}$.For evaluation purpose only, temporary hard cluster labels are derived:


$$\begin{array}{c} z_{i}={argmax}_{1\leq j\leq k}U_{ij} \end{array}$$ (8)

where $U_{ij}$ denotes the fuzzy membership of sample i belonging to cluster j. The silhouette score S(k) is computed to evaluate the clustering quality under candidate k.


$$\begin{array}{c} k^{*}={argmax}_{k\in \{2,\ldots ,8\}}S\left(k\right) \end{array}$$ (9)

After the optimal $k^{*}$ is determined, FCM is re‑executed with $k^{*}$ to obtain the final fuzzy‑membership matrix for subsequent oversampling operations.

#### iv) Standard FCM algorithm steps.

Input: Data $X\in R^{N\times d}$, number of clusters C, fuzzifier m>1, convergence threshold ε, and maximum iteration T.

Output: Cluster centers $\left\{c_{j}\right\}$ and membership matrix 𝐔.

Initialize the membership matrix ${𝐔}^{\left(0\right)}$: it can be randomly initialized such that, for each sample, the memberships across all clusters sum to 1; alternatively, initialize the centers at random and then compute the initial memberships.

Repeat the following steps:

(t=0,1,2, Until convergence or reaching…T):

Update cluster centers


$$\begin{array}{c} c_{j}^{\left(t+\text{1}\right)}=\frac{\sum_{i=\text{1}}^{N}{\left(u_{ij}^{\left(t\right)}\right)}^{m}x_{i}}{\sum_{i=\text{1}}^{N}{\left(u_{ij}^{\left(t\right)}\right)}^{m}} \end{array}$$ (10)

Compute the distance from each sample to every cluster center


$$\begin{array}{c} d_{ij}=\left\|x_{i}-c_{j}^{\left(t+\text{1}\right)}\right\| \end{array}$$ (11)

(In implementation, a small smoothing term is typically applied when $d_{ij}=\text{0}$ to avoid division by zero.)

Update membership degrees


$$\begin{array}{c} u_{ij}^{\left(t+\text{1}\right)}=\frac{\text{1}}{\sum_{k=\text{1}}^{C}{\left(\frac{d_{ij}}{d_{ik}}\right)}^{\frac{\text{2}}{m-\text{1}}}} \end{array}$$ (12)

Compute the objective function


$$\begin{array}{c} J_{m}^{\left(t+\text{1}\right)}=\sum_{i=\text{1}}^{N}\sum_{j=\text{1}}^{C}{\left(u_{ij}^{\left(t+\text{1}\right)}\right)}^{m}d_{ij}^{\text{2}} \end{array}$$ (13)

Convergence check


$$\begin{array}{c} {max}_{i,j}\left|u_{ij}^{\left(t+\text{1}\right)}-u_{ij}^{\left(t\right)}\right|<\varepsilon \end{array}$$ (14)

$$\begin{array}{c} \text{or\textasciitilde{}}\left|J_{m}^{\left(t+\text{1}\right)}-J_{m}^{\left(t\right)}\right|<\varepsilon \end{array}$$ (15)

f satisfied, stop; otherwise, proceed to the next iteration.

Return: the final cluster centers $\left\{c_{j}\right\}\;$and the membership matrix U

As shown in Table 1, this is the pseudocode for performing SMOTE sampling on the dataset based on fuzzy clustering.

**Table 1:** Steps of the membership-guided FCM-SMOTE algorithm.

Input: X, y, n_clusters, m, k_neighbors, same_cluster_weight, min_cluster_size, seed
Output: X_out, y_out
1. Count the number of samples per class: counts，max_count = max(counts)
2. Initialize X_res = [X], y_res = [y], Set the random seed rng(seed)
3. For each class cls:
if counts[cls] >= max_count: continue
need = max_count - counts[cls]
X_cls = {x | y==cls}
if len(X_cls) < 2: continue
U, labels = FCM(X_cls, c=n_clusters, m=m) (Obtain cluster labels from FCM)
knn_global = kNN_fit(X_cls, n_neighbors=min(k_neighbors, len(X_cls))) (Class-wide kNN (fallback))
For each cluster k:
idx_k = {i | labels[i]==k}
if |idx_k| >= min_cluster_size:
knn_cluster[k] = kNN_fit(X_cls[idx_k], n_neighbors=min(k_neighbors, |idx_k|))(kNN within each cluster)
Compute membership-guided sampling weights:
For each sample i:
m_i = max_k(u_ik)
w_i = 1 - m_i
Calculate sampling probability:
p_i = exp(βw_i) / Σ exp(βw_j)
repeat need times:
i ~ Categorical(p1,p2,...,pN); xi = X_cls[i]; ci = labels[i]
use_same = (ci ∈ knn_cluster) AND (rng.rand() < same_cluster_weight)
nn_pool = neighbors_from(knn_cluster[ci])
if use_same
else neighbors_from(knn_global)
j ~ Uniform(nn_pool); xj = X_cls[j]
λ = exp(βw_i)/(exp(βw_i)+exp(βw_j)); x_new = xi + λ*(xj - xi)
Append x_new to the synthetic set with label cls
(Synthesize need samples)
Append the synthetic set to X_res, y_res
4) X_out = concat(X_res), y_out = concat(y_res); return.

### (3) Extracting higher-order features with deep learning

This paper proposes a three-branch parallel–fusion feature extraction architecture for intrusion detection in the Industrial Internet of Things. Taking reordered tabular‑derived traffic features as input, the model activates three complementary branches:① Spatial branch: employs Pyramid Depthwise Separable Convolutions (PDSC) to hierarchically capture local and multi‑scale spatial patterns;②Temporal branch: uses a BiGRU to learn forward–backward dependencies and long‑range temporal relations from real time‑ordered traffic sessions; ③ Frequency branch: applies a wavelet transform to extract multi‑scale spectral‑statistical features from an artificially constructed computational dimension.The high-level representations from the three branches are concatenated along the channel dimension and fed into a fusion head (Dropout → Fully Connected → ReLU → Dropout → Fully Connected) for feature reweighting and discrimination, after which a Softmax layer outputs probabilities over N classes. This design achieves information complementarity and noise suppression across the spatial, temporal, and frequency domains, improving the separability of multiple attack types and the model’s robustness without significantly increasing computational overhead.

#### ⅰ) Spatial feature extraction via parallel pyramid depthwise separable convolutions.

a. From features to a grid representation. As shown in Fig 1, for a traffic sample we reorganize its numerical feature vector$\;𝐟\in R^{d}$ (in CIC-IDS2017, d≈78) into a tensor ${X\in R}^{C\times H\times W}$, with the constraint H·W=d. To match the input dimensionality expected by subsequent convolutional networks, we first increase the channel dimension to C=64. In this representation, the vertical dimension H corresponds to “time slices/segments,” while the horizontal dimension W arranges the various statistics and flags within each time slice. In this way, tabular features are transformed into a “pseudo-image” with local adjacency, enabling convolutional networks to learn cross-feature collaborative patterns within spatial neighborhoods (Fig 2).

**Fig 1:**
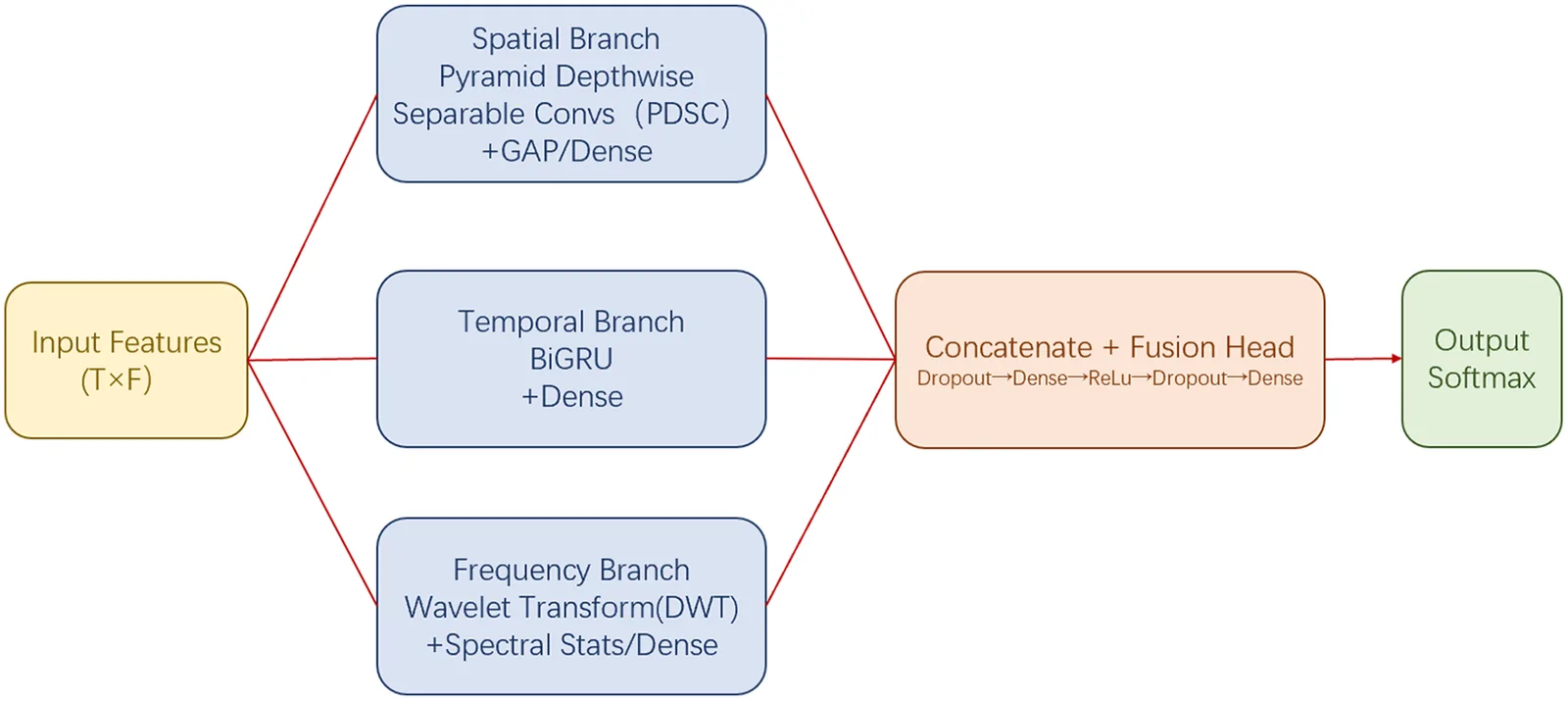
Three-branch parallel fusion architecture for IIoT intrusion detection (PDSC–BiGRU–Wavelet).

**Fig 2:**
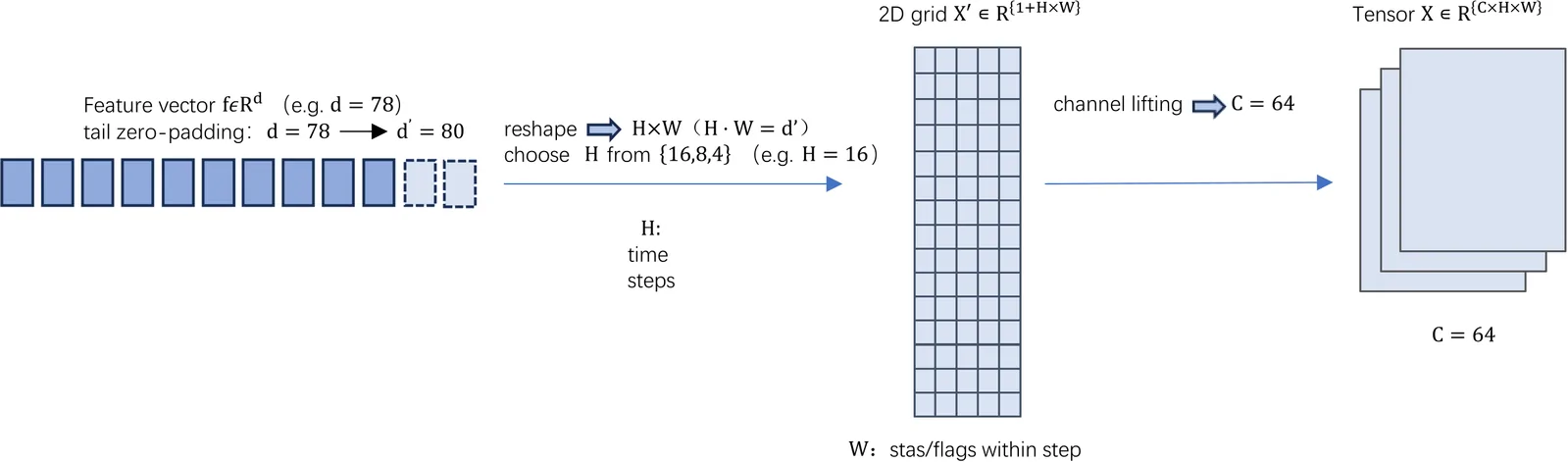
Feature-to-grid representation.

b. Parallel Pyramid Depthwise Separable Convolution (PDSC). Capture short-, medium-, and longer-range correlations along the horizontal (feature) dimension—while keeping parameters and computation modest—and fuse the scales in a learnable manner.

Given an input feature map $X\in R^{C\times H\times W}$. The module consists of mmm parallel depthwise branches (here m=3 with kernel sizes{3,5,7}), Each branch performs convolution only along the horizontal axis with a 1×k  kernel while keeping the spatial size unchanged.The branch outputs are then concatenated along the channel dimension, followed by a 1×1convolution to achieve cross-channel fusion and reduce the channels back to the original count. Batch Normalization (BN) and ReLU are applied to each branch and to the fusion layer. Formally:


$$\begin{array}{c} U_{k}=ReLu(BN(DW_{\text{1}\times k}\;(X))),k\in \left\{\text{3},\text{5},\text{7}\right\}, \end{array}$$ (16)

$$\begin{array}{c} \tilde{U}=Concat_{ch}\left(U_{\text{3}},U_{\text{5}},U_{\text{7}}\right)\in R^{(\text{3}C)\times H\times W}, \end{array}$$ (17)

$$\begin{array}{c} Y=ReLu\left(BN\left(Conv_{\text{1}\times \text{1}}\left(\tilde{U}\right)\right)\right)\in R^{C\times H\times W}. \end{array}$$ (18)

Here, $DW_{\text{1}\times k}$ denotes a depthwise convolution (groups=C), and the padding is set to $(\text{0},\left\lfloor \frac{k}{\text{2}}\right\rfloor )\;$to keep W unchanged; $Conv_{\text{1}\times \text{1}}$ performs channel mixing and dimensionality reduction.Let the set of branch kernels be ${\left\{k_{i}\right\}}_{i=\text{1}}^{m}$. Ignoring the statistics and affine parameters of BN, the number of convolutional layers is


$$\begin{array}{c} \underset{depwise}{\underbrace{\sum_{i=\text{1}}^{m}C\cdot k_{i}}}\;+\;\underset{\text{1}\times \text{1}\text{ fusion}}{\underbrace{mC^{\text{2}}}}\;. \end{array}$$ (19)

For example, with $C=\text{6}\text{4},\left\{k_{i}\right\}=\left\{\text{3},\text{5},\text{7}\right\},m=\text{3}$:

DW parameters =64· (3+5+7) =960;

1×1 parameters =3·64^2^=12,228,

total≈13,248.

The main computational cost lies in the 1 × 1 fusion, but compared with a standard convolution offering the same receptive field (parameters~$C^{\text{2}}\cdot k$), it still achieves a substantial reduction in parameters and computation.

Its representational properties include: Multi-scale coexistence: the three kernel sizes are sensitive to short-range (1 × 3), mid-range (1 × 5), and longer-range (1 × 7) horizontal dependencies, respectively; the parallel design preserves responses at each scale rather than mixing and diluting them within a single kernel. Learnable fusion: the 1 × 1 convolution performs linear recombination along the channel dimension, effectively weighting, selecting, and combining multi-scale evidence so the network can adaptively prefer the most effective scale(s). Anisotropic design: convolution is applied only along W, focusing on relationships among different statistics within the same time step, while cross-step information along H is aggregated by subsequent pooling and global average pooling (GAP). As shown in Fig 3, this is a module-level schematic of the Parallel Pyramid Depthwise Separable Convolution (PDSC) module.

**Fig 3:**
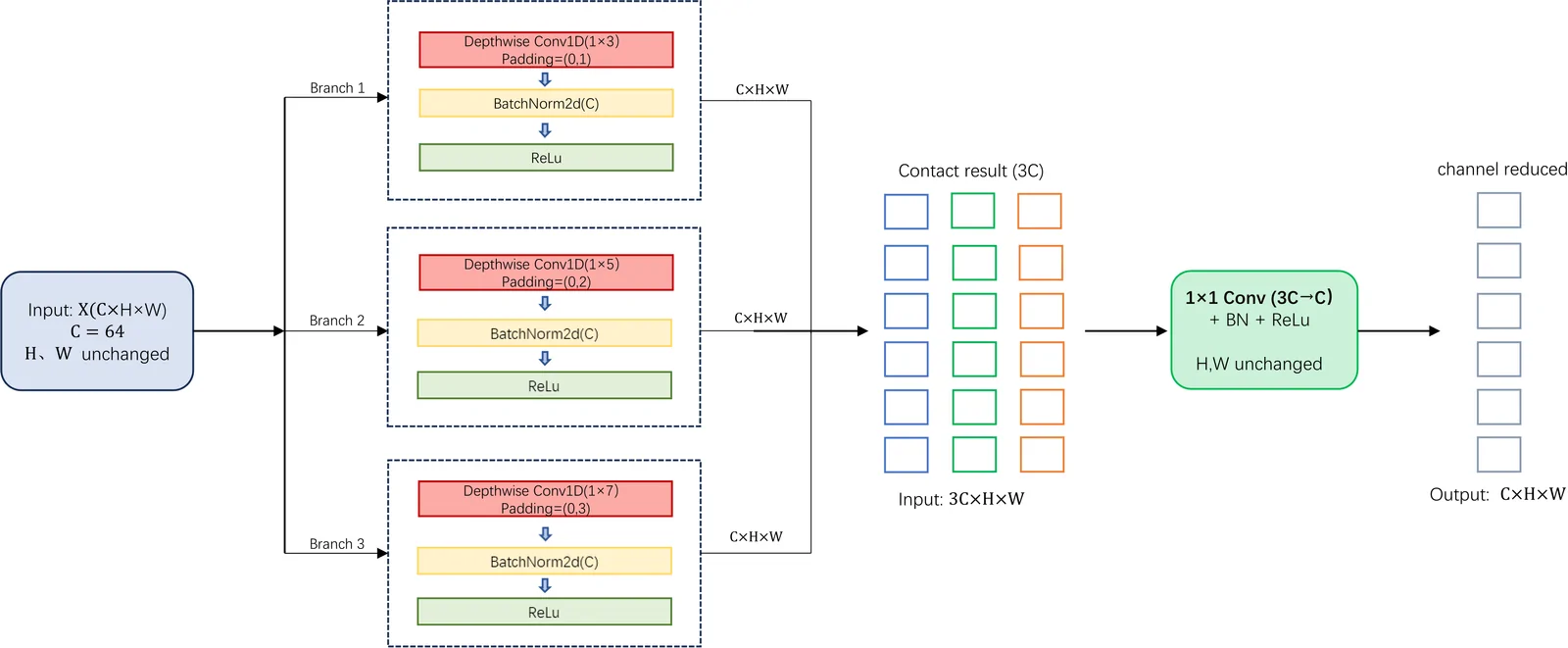
PDSC module (Module-level magnified diagram).

c. Spatial Branch Network and Integration. In the overall model (Spatial Branch), we cascade two PDSC blocks and, after each PDSC, append a 1×1 convolution (for channel re-mixing) and 2×2 max pooling to enhance scale invariance and robustness. We then apply global average pooling (GAP) followed by two fully connected layers to obtain the class logits $𝐥𝐨𝐠𝐢𝐭𝐬\;\hat{y}$:


$$\begin{array}{c} \hat{y}=FC_{\text{2}}(ReLU(FC_{\text{1}}(GAP(Z)))). \end{array}$$ (20)

The loss function is the multi-class cross-entropy


$$\begin{array}{c} L=CE(\hat{y},y), \end{array}$$ (21)

During training, the logits are used directly; during inference, a softmax is applied to obtain posterior probabilities. As shown in Fig 4, this is the architecture of the spatial feature extraction branch.

**Fig 4:**
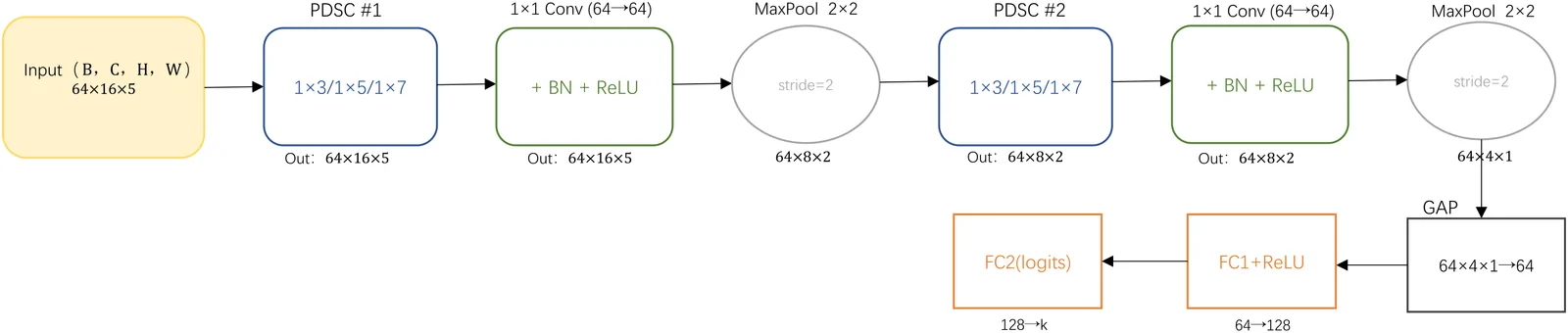
Architecture of the spatial feature extraction branch.

#### ⅱ) BiGRU for temporal feature extraction.

As shown in Fig 5, this is the architecture of the BiGRU sequence-level classification model. From the dataset, sessions or fixed time windows are aggregated and ordered chronologically to form a sequence $X\in R^{T\times d}\;$of length T  (with d=39 in our experiments). Z-score normalization is applied using statistics computed from the training set. A single-layer bidirectional GRU (hidden size H) is used, producing a forward state ${\vec{h}}_{t}$ and a backward state ${\overset{\leftarrow }{h}}_{t}$ at each time step. The gating mechanism preserves long-term dependencies while suppressing noise.

**Fig 5:**
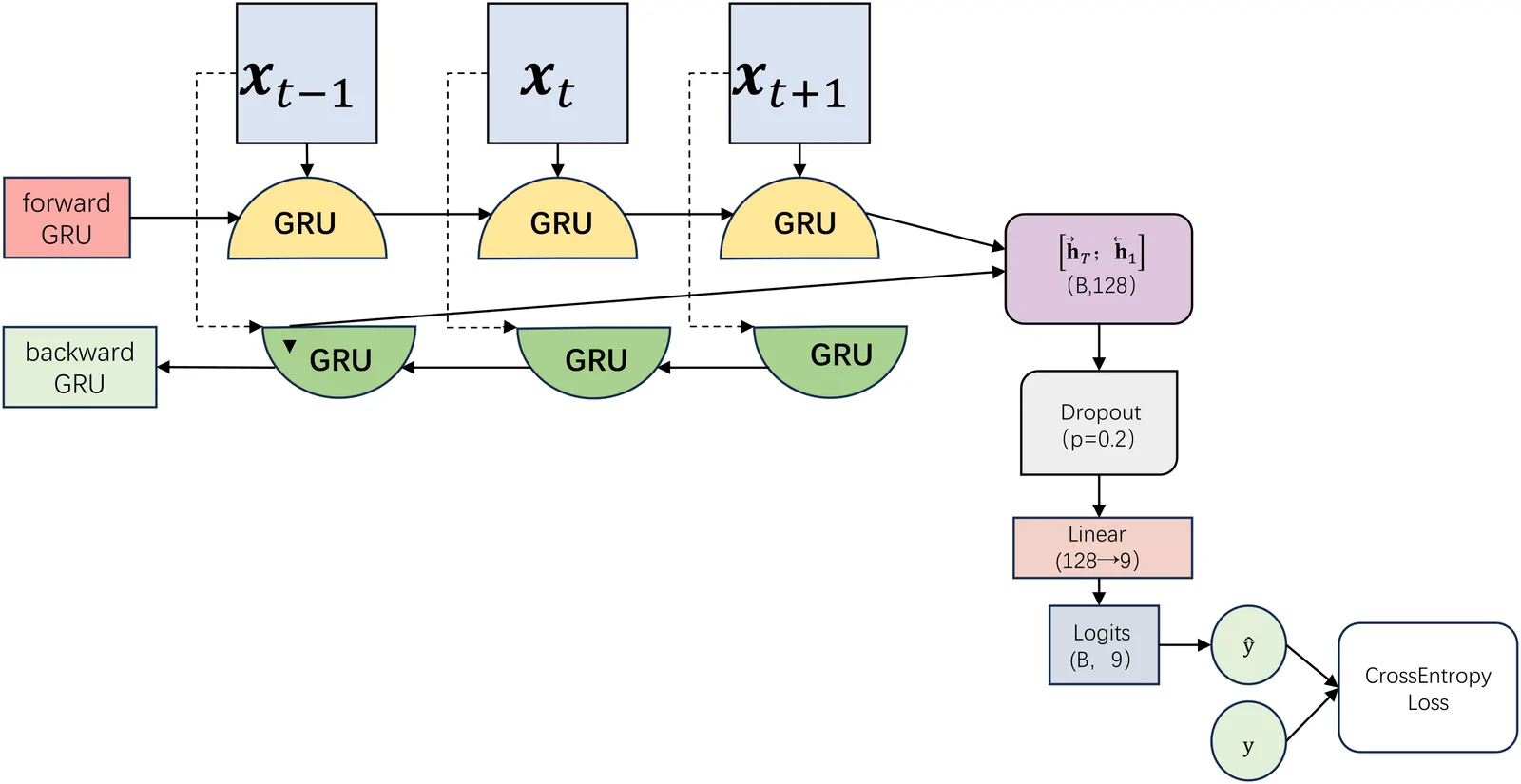
Architecture of the BiGRU-based sequence-level classifier.

Concatenate the final forward hidden state and the final backward hidden state to obtain a global representation.


$$\begin{array}{c} h_{*}=\left[{\vec{h}}_{T};{\overset{\leftarrow }{h}}_{\text{1}}\right]\in R^{\text{2}H}. \end{array}$$ (22)

After $h_{*}$, apply Dropout (p=0.2) ollowed by a linear layer to produce C- dimensional logits (in our experiments C=9). Use the cross-entropy loss:


$$\begin{array}{c} L=-\frac{\text{1}}{N}\sum_{i=\text{1}}^{N}\sum_{c=\text{1}}^{C}\text{1}\left(y_{i}=c\right)log\;(softmax{\left(o_{i}\right)}_{c}). \end{array}$$ (23)

BiGRU elevates single-step statistics into cross-temporal behavioral representations, and can simultaneously encode rate, rhythm, event order, and directional asymmetry. Through its bidirectional structure, it captures global (past and future) context, while gated memory highlights long-range dependencies. As a result, the model achieves greater separability when distinguishing traffic with similar static features but different dynamic patterns.

#### ⅲ) Wavelet spectral branch for traffic.

a. Sequence construction and feature normalization

Starting from CICIDS2017 tabular traffic records, we reorganize static tabular samples into ordered traffic sequences with a fixed time-window arrangement to obtain traffic sequences of length T:


$$\begin{array}{c} X=\left[x_{\text{1}},...,x_{T}\right]\text{\textasciitilde{}}\in \text{\textasciitilde{}}R^{T\times d},\text{\textasciitilde{}}x_{t}\in R^{d}. \end{array}$$ (24)

Compute the normalization parameters (μ,σ) on the training set over the flattened (B·T) × d dimension, and apply them to the training, validation, and test sets:


$$\begin{array}{c} {\hat{𝐗}}_{t}=({𝐗}_{t}-\mu )⊘\sigma . \end{array}$$ (25)

To interface with the wavelet branch, the input needs to be rearranged from time-major layout (B,T,d) to channel-first layout (B,C,T) where C=d. (Here, B is the batch size; T: is the sequence length in time steps; d is the feature dimension at each time step; and C denotes the number of channels—each feature is treated as an independent channel, hence C=d.) The wavelet branch executes one-dimensional discrete wavelet transform along the time dimension T for each independent traffic-feature channel. Using the (B,C,T) channels-first layout facilitates channel-wise parallel processing in implementation. This rearrangement changes only the axis order, not the numerical values.

*Note that the one‑dimensional dimension constructed for DWT decomposition is only for computational implementation. It does not correspond to the real chronological evolution of network traffic and is solely used as a computational carrier to realize multi‑scale statistical decomposition of static tabular flow features, rather than performing time‑frequency analysis for time‑varying signals.*

b. Within-channel discrete wavelet transform

Perform a discrete wavelet transform on each channel’s computational‑dimension feature vector of length T using the Daubechies‑1 wavelet as the mother wavelet. The desired decomposition level is set to $L_{req}=\text{3}$, the actual effective level is determined by the sequence-length constraint:


$$\begin{array}{c} L=min(L_{req},\left\lfloor {\text{log}}_{\text{2}}T\right\rfloor ). \end{array}$$ (26)

The DWT produces a first-level approximation subband and successive detail subbands at each level$\left\{A_{L},D_{L},\ldots ,D_{\text{1}}\right\}$, In this setting with T=2, the effective level is L=1, yielding$\left\{A_{\text{1}},D_{\text{1}}\right\}$ for each channel.

c. Subband statistical features

For each subband’s coefficients$\;{\left\{w_{i}\right\}}_{i=\text{1}}^{n}$ compute two types of statistics: energy and entropy:


$$\begin{array}{c} energy:E=\sum_{i=\text{1}}^{n}w_{i}^{\text{2}}, \end{array}$$ (27)

$$\begin{array}{c} entropy:p_{i}=\frac{w_{i}^{\text{2}}}{\sum_{j}w_{j}^{\text{2}}+\varepsilon },\text{\textasciitilde{}}H=-\sum_{i=\text{1}}^{n}p_{i}\text{log}(p_{i}+\varepsilon ). \end{array}$$ (28)

Apply logarithmic compression to the energy to reduce dynamic range. Let the number of statistics be S=2 (energy and entropy), Then the feature dimension per channel is (L+1)×S, and the spectral feature dimension for the whole sample is:


$$\begin{array}{c} D_{\text{feat}}=C(L+\text{1})S. \end{array}$$ (29)

When C=39,L=1,S=2, the feature dimension is $D_{\text{feat}}=\text{1}\text{5}\text{6}$.

Feed the concatenated vector in $R^{D_{feat}}\;$into a lightweight fully connected classification head to output K-class logits:


$$\begin{array}{c} 𝐨=𝐖\;𝐟\;+𝐛\;\in R^{K}, \end{array}$$ (30)

Where $𝐟\in {\text{R}}^{D_{feat}}$ is the spectral statistical representation, and K is the number of classes (in our experiments K=7 or dataset-dependent). End-to-end optimization is performed using weighted cross-entropy (class weights obtained via a frequency-balancing strategy on the training set). The optimizer is Adam, and the learning rate is adaptively decayed by a Reduce-on-Plateau scheduler using validation loss as the criterion. At the first forward pass, the model dynamically determines $D_{feat}$ based on the actual T and L and constructs the classification head accordingly, thereby naturally accommodating different sequence lengths and decomposition levels.

This method adopts a “channel‑wise DWT + statistical pooling” paradigm to mine time‑frequency statistical patterns from reordered tabular‑derived computational‑dimension samples. It explicitly quantifies the magnitude and uncertainty of low‑frequency stable statistical trends and high‑frequency statistical fluctuations reflected in network traffic behaviours. Even for extremely short computational‑dimension samples (T=2), it provides each original traffic feature with a dual‑scale statistical perspective of “variation versus steady state”. As the computational‑dimension length increases, more feasible decomposition levels become available and subband diversity grows accordingly, further improving the discriminative capability of the learned spectral‑statistical representation. (As shown in Fig 6: the architecture of the wavelet spectral branch.)

**Fig 6:**
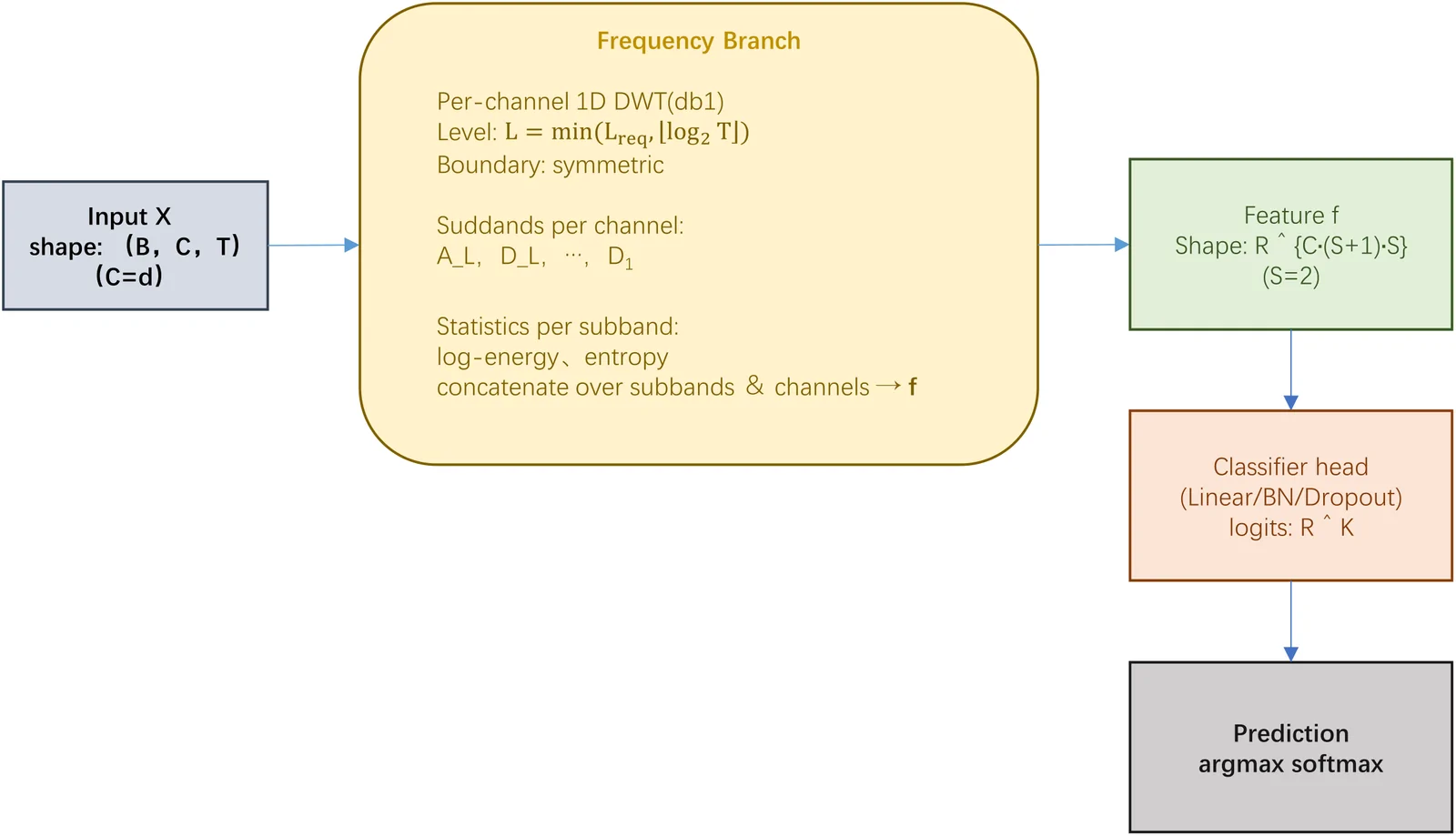
Architecture of the wavelet-spectral branch model.

### (4) To further clarify the implementation of the proposed three‑branch network and improve experimental reproducibility, the detailed layer‑wise configurations are summarized as follows

#### Network architecture details.

The proposed three‑branch parallel network consists of spatial‑domain, temporal‑domain, and frequency‑domain branches, followed by a weighted attention‑based feature‑fusion layer for multi‑dimensional traffic feature learning.

For the spatial‑domain branch, two sequential one‑dimensional convolutional layers (Conv1D) are adopted to extract local spatial patterns from pre‑processed network‑traffic sequences. The first convolution layer employs 64 filters with kernel size of 3 and stride of 1, followed by ReLU activation and batch normalization. The second Conv1D layer uses 32 filters with kernel size of 3 and stride of 1, also coupled with ReLU activation and batch normalization. The output feature dimension of the spatial branch is set to 32.

The temporal‑domain branch utilizes stacked two‑layer GRU units to capture long‑range time‑dependent characteristics of traffic samples. Each GRU layer contains 48 hidden‑state neurons. Dropout with a rate of 0.2 is applied after each GRU layer to mitigate overfitting risks. The final output hidden state of the second GRU layer is taken as the temporal feature representation, yielding a feature dimension of 48.

In the frequency‑domain branch, discrete wavelet transform is first performed to obtain multi‑scale time‑frequency statistical coefficients. Afterwards, a single‑layer 1‑D convolution block with 32 filters (kernel size = 3) and ReLU activation is used to further abstract frequency‑domain high‑level features, producing output features with dimension 32.

After obtaining feature representations from three individual branches, an attention‑weighted fusion layer is leveraged to assign learnable importance weights to spatial, temporal and frequency‑domain features respectively. The weighted features are concatenated and fed into two fully‑connected layers. The first dense layer contains 64 neurons with ReLU activation; the final output dense layer uses softmax activation for multi‑class intrusion‑classification prediction, whose output dimension corresponds to the total number of traffic categories in the CICIDS2017 dataset.

#### Hyper‑parameter tuning mechanism.

In this work, we adopt a validation‑set‑driven grid‑search strategy for hyper‑parameter selection. The original training dataset is randomly split into training subsets and validation subsets at a ratio of 8:2. The validation subset is strictly isolated and never involved in model training process.

Key hyper‑parameters including initial learning rate, dropout rate, batch size, and the number of neurons within GRU and fully‑connected layers are searched within predefined candidate ranges:

1) Initial learning rate: {1e‑4, 5e‑4, 1e‑3, 5e‑3};
2) Dropout rate: {0.1, 0.2, 0.3, 0.4};
3) Batch size: {16, 32, 64, 128};
4) GRU hidden‑neuron count: {32, 48, 64};
5) Dense‑layer neuron count: {32, 64, 128}.

For each hyper‑parameter combination, the model is trained on the training subset and evaluated on the held‑out validation subset. The G‑mean metric on the validation set is taken as the core optimization indicator, since G‑mean is insensitive to class imbalance. The hyper‑parameter group achieving the highest validation‑set G‑mean score is selected as the final configuration for subsequent formal testing on the unseen test set.

Moreover, the Adam optimizer is employed for network weight updating. Early‑stopping strategy is enabled: if validation‑set G‑mean does not improve for 12 consecutive training epochs, the training procedure will terminate in advance to prevent over‑fitting. The maximum training epoch is set to 100.

### (5) Computational complexity analysis

The FCM‑driven adaptive oversampling acts purely as a training‑set preprocessing procedure and is fully decoupled from online intrusion‑detection inference. The time complexity of the FCM clustering procedure is $O\left(N\cdot c^{\text{2}}\cdot t\right)$, where N denotes the number of minority‑class samples, c is the predefined cluster number, and t represents the number of iteration rounds. This clustering‑resampling step is executed only once on the training dataset before model training, and it will not be invoked during the testing or deployment phase. For the proposed three‑branch parallel network, the inference‑stage time complexity is dominated by Conv1D and GRU operations, which scales with the input‑sequence length and model parameter size. Only neural‑network forward propagation is performed for incoming traffic samples during real‑time detection, so the online computational overhead remains acceptable for IIoT intrusion‑detection tasks.

It is worth noting that this study focuses on multi‑branch network architecture and fuzzy‑clustering‑assisted resampling for imbalanced intrusion detection, rather than proposing novel feature selection algorithms. Some well‑known SOTA feature‑selection approaches (e.g., mutual‑information‑based filtering, ReliefF, L1‑regularized embedded selection) have been widely applied in intrusion‑detection tasks to remove redundant features. Comprehensive experimental benchmarking against these approaches falls outside the core scope of this study and will be pursued in our follow‑up research dedicated to feature optimization.

## 4 Results and discussion

### (1) Datasets

This study uses the real-world cybersecurity dataset **CICIDS2017**, which covers seven categories of network traffic: Normal, Denial of Service (DoS), Distributed Denial of Service (DDoS), PortScan, Patator (password-guessing attacks), Web Attack, and Bot. DoS attacks continuously send excessive packets to a target, blocking normal access; PortScan scans a small set of ports across many hosts or traverses many ports on a single host; Patator performs online password guessing against multi-protocol authentication interfaces; DDoS coordinates numerous compromised hosts to transmit packets simultaneously, exhausting bandwidth and compute resources; Web Attack is a set of attacks targeting web applications (e.g., SQL injection, brute force); and Bot refers to a host controlled by malicious software.

The respective sample sizes for these seven classes are 227309 (Normal), 44706 (DoS), 15929 (DDoS), 13835 (PortScan), 12802 (Patator), 2180 (Web Attack), and 1966 (Bot).The detailed per‑class sample distribution is summarized in Table 2.

**Table 2:** Class‑wise sample distribution of the CICIDS2017 dataset.

class	Normal	DoS	DDoS	PortScan	Patator	Web Attack	Bot
Number of samples	227309	44706	15929	13835	12802	2180	1966

### (2) Experiment settings

This study formulates abnormal traffic detection as a multi-class classification problem: the model takes network traffic as input and outputs its corresponding traffic type. We split the entire dataset into training and test sets in a 7:3 ratio—the former is used to learn model parameters, and the latter to evaluate generalization. To enable an intuitive comparison across models, we adopt four common evaluation metrics: **Accuracy**, **Precision**, **Recall**, and **F-Score**.

### (3) Results analysis

#### ⅰ) FCM-SMOTE-boosted.

This paper employs a boundary-enhanced oversampling method based on Fuzzy C-Means and evaluates its impact on class distribution and data geometry using the CIC-IDS2017 training set. Figs 8–10 respectively present comparisons of class counts, PCA visualizations, and feature distributions before and after oversampling.

a. Category distribution

Before oversampling (training set distribution):Normal 1,818,477; DoS 202,137; PortScan 127,173; DDoS 102,422; Patator 11,068; Web Attack 1,744; Bot 1,573.

Target counts after oversampling:Normal 1,818,477 (kept at 1.0×); DoS 676,675 (≈3.35×); PortScan 549,309 (≈4.32×); DDoS 498,329 (≈4.87×); Patator 66,408 (6×); Web Attack 10,464 (6×); Bot 9,438 (6×).

After oversampling, the total sample size is about 3.63M. The majority class (Normal) accounts for roughly 50%, which is markedly lower than in the original data; rare classes (Web Attack / Bot / Patator) are increased by orders of magnitude while still maintaining a “non-uniform yet controlled” structure consistent with the real distribution. Under the strict constraint that the majority-class multiplier = 1.0, the method effectively improves minority-class representativeness, and—with a proportion threshold—avoids distribution shift and excessive training costs that could result from “over-balancing.” (Fig 7).

**Fig 7:**
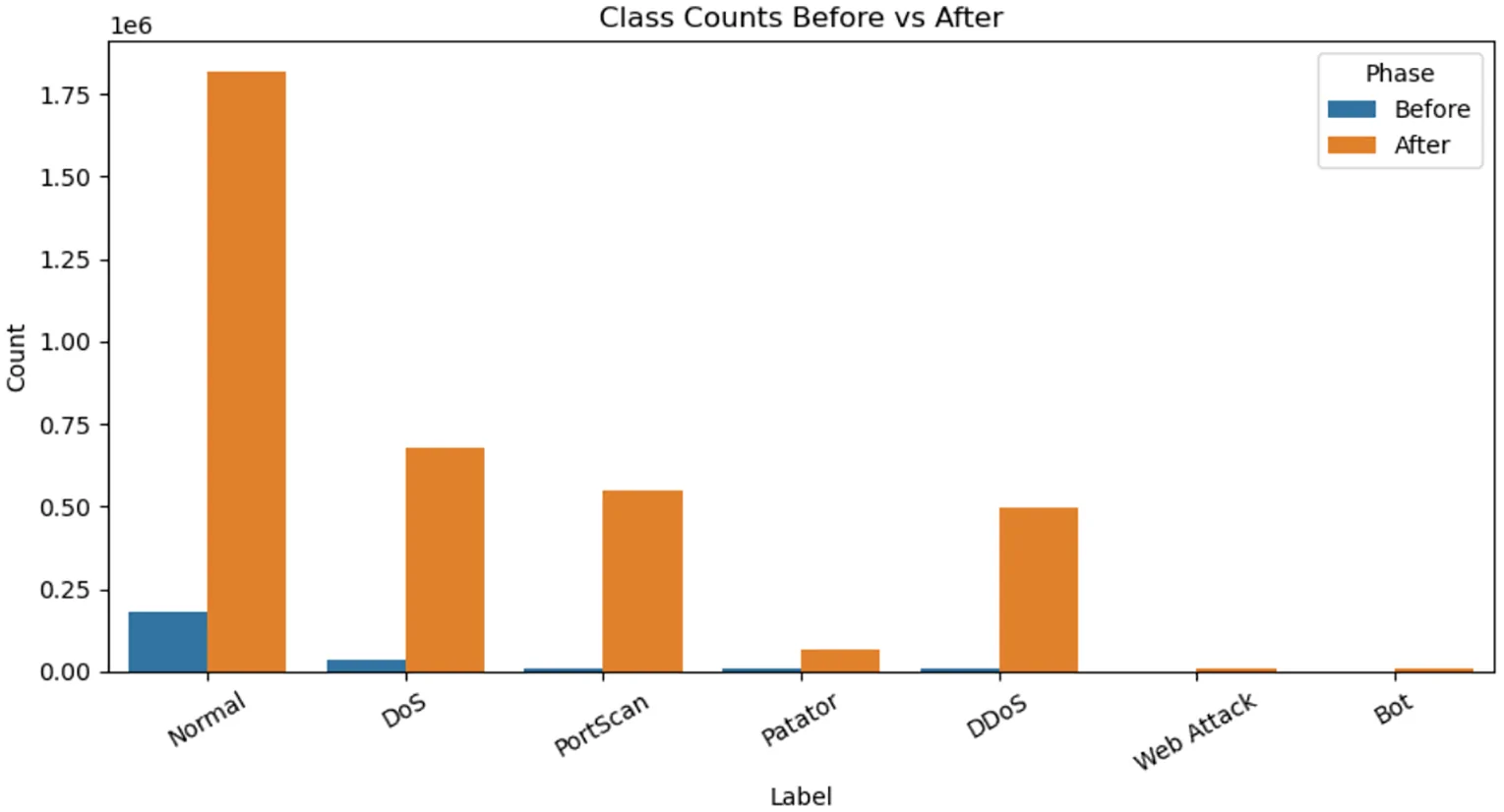
Class counts Before vs After.

b. Geometric structure of the data

The After (×) distribution essentially unfolds along the manifold of Before (●), with no large-scale drift or emergence of new outlier patterns, indicating that the synthetic samples follow the low-dimensional structure of the original data. In addition, a certain radial/striated texture can still be observed—this is a typical trace of neighborhood interpolation. However, thanks to the use of “multi-neighbor convex combinations + membership-weighting + slight jitter,” the stripes are finer and more continuous, with no sizable anomalous clusters or mode collapse.

For classes such as DDoS/DoS, the density near inter-class boundaries increases noticeably in the After distribution, which is expected to strengthen decision boundary learning and improve minority-class recall. Overall, oversampling primarily “densifies” samples along the existing manifold and within boundary neighborhoods, keeping the geometric structure stable; the method achieves boundary enhancement rather than mode expansion (Fig 8).

**Fig 8:**
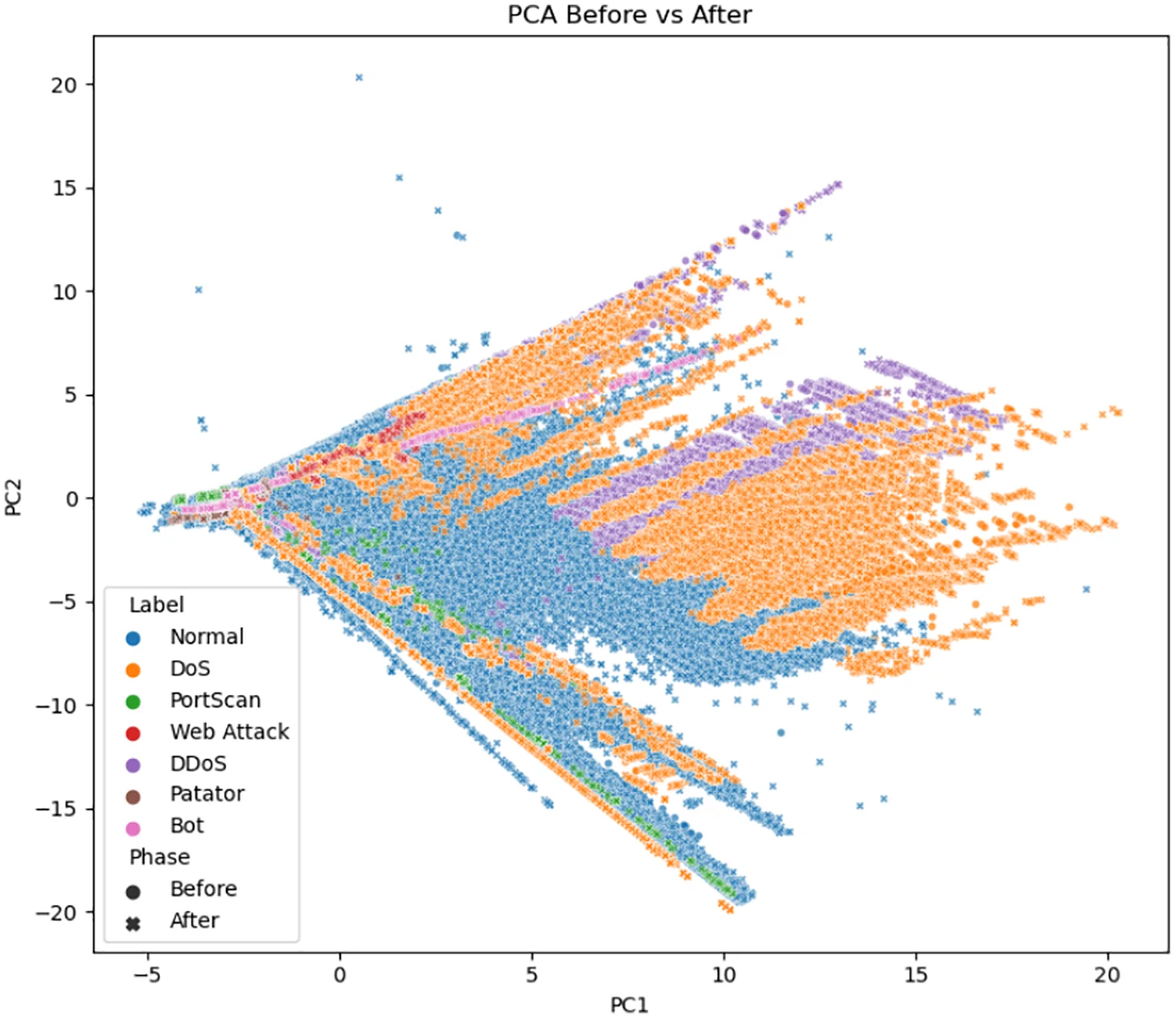
PCA Before vs After.

c. Feature marginal distributions

The positions of the main peaks and the tail distributions are largely consistent before and after; the **After** peaks are slightly sharper with higher density, consistent with the expectation that “more interpolated samples are generated in high-density regions.” No abnormal accumulation is observed in the long-tail or extreme ranges, indicating minimal shift in marginal distributions. The method increases local density while maintaining consistency of key feature statistics, and no significant marginal distortion is observed (Fig 9).

**Fig 9:**
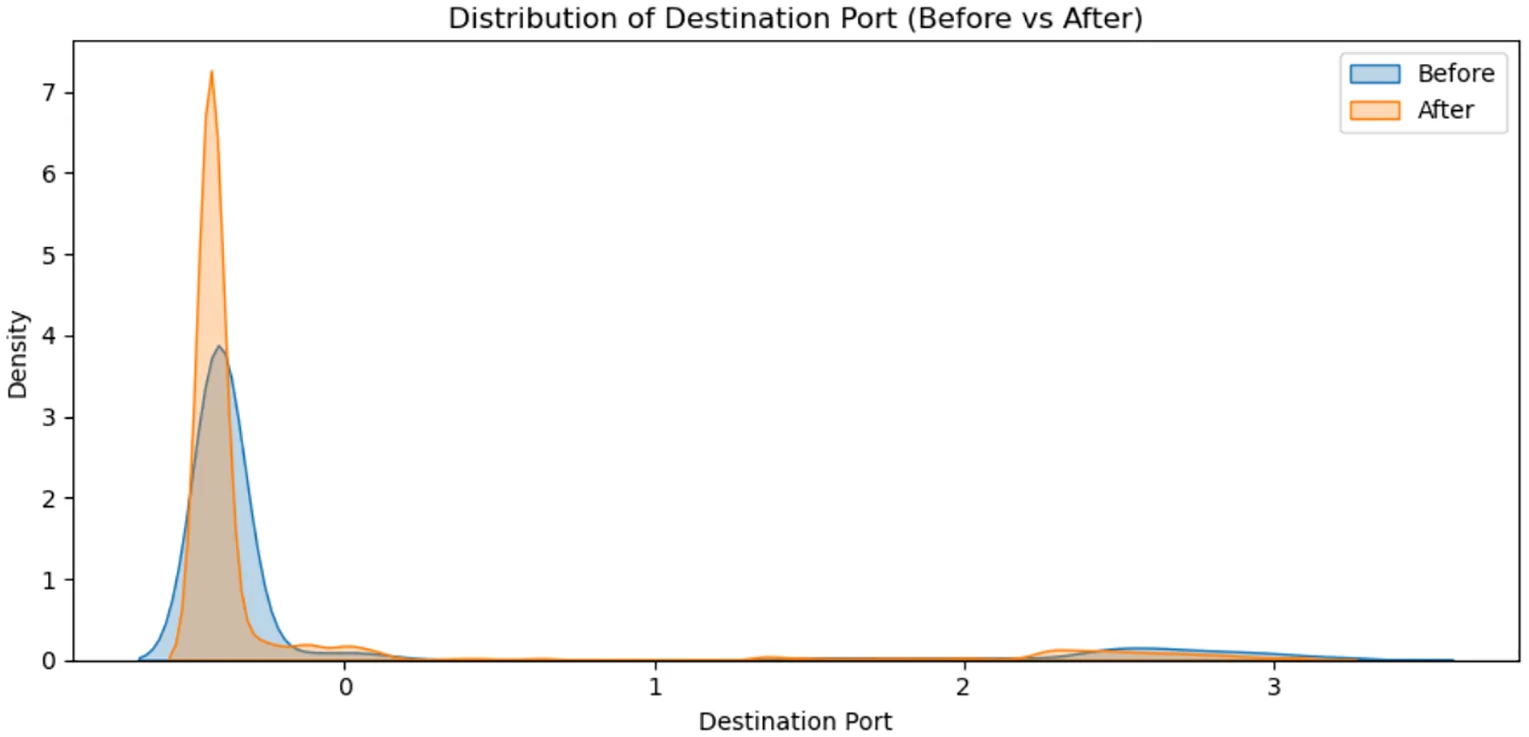
Distribution of destination port (Before vs After).

#### ⅱ) Multi-class classification task.

a. Before sampling

As shown in Fig 10, from left to right are the results of CNN, CNN-LSTM, BiGRU, and the proposed model (FPBW-IDS) on the multiclass task before any oversampling of the dataset. Observations indicate that FPBW-IDS outperforms the other three models across the attack types. The model not only predicts a larger number of samples correctly for the majority class “Normal” than the others, but for the extremely rare class Bot it performs second only to CNN—yet still better than the other two models.

**Fig 10:**
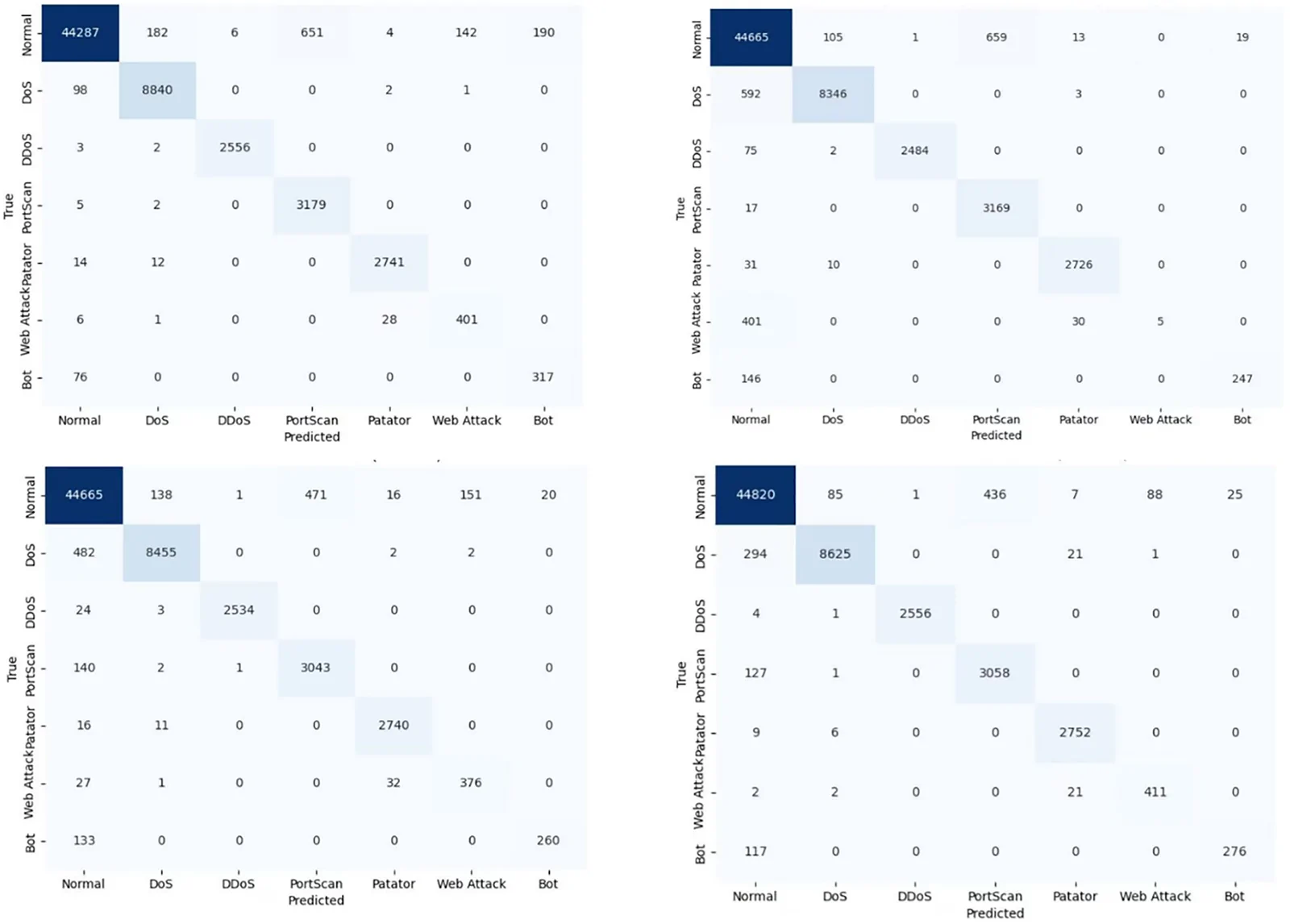
Confusion Matrix for Multi-Class Classification(CNN, CNN-LSTM, BiGRU, FPBW-IDS).

To investigate the capability of different models to learn representative IIoT traffic patterns, we conduct comparative experiments with three baseline models including a spatial‑oriented CNN, a temporal‑oriented LSTM, and a naive CNN+LSTM fusion model, alongside our proposed FPBW‑IDS. Training and validation accuracy curves within 100 training epochs are recorded and visualized in Fig 11.

**Fig 11:**
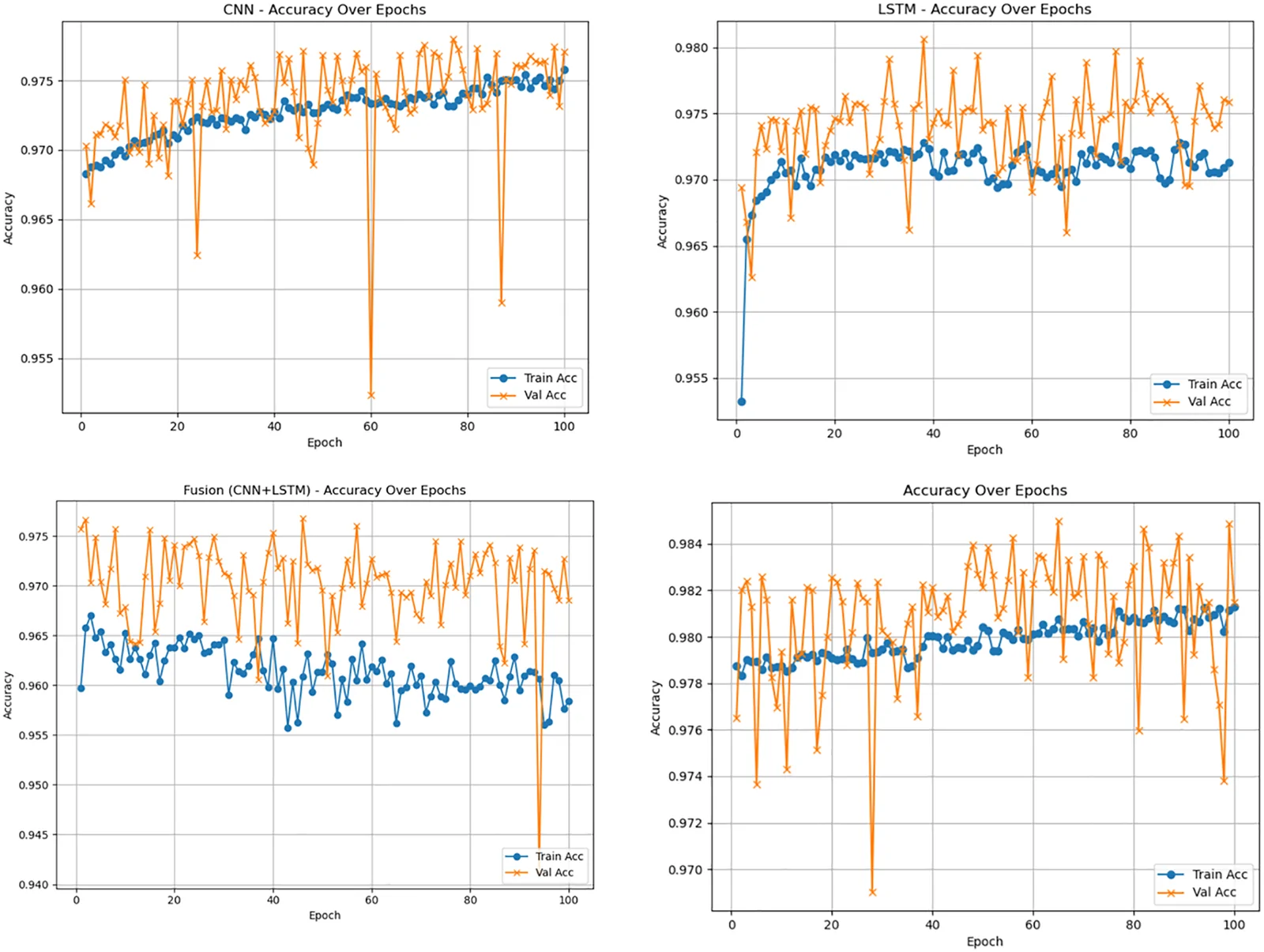
Comparison of accuracy over Epochs among CNN, LSTM, Fusion, and Ours.

**CNN:** Training accuracy rises smoothly from ~96.8% to ~97.5%; validation accuracy is generally on par with the training curve. **LSTM:** Training accuracy converges quickly to ≈97.0–97.3%; validation accuracy is overall higher (≈97.3–98.0%) with slightly larger fluctuations. **CNN+LSTM (fusion baseline):** Training accuracy is about 95.5–96.5%, clearly lower than the first two; validation accuracy stays around ≈96.8–97.5% with occasional dips (early fusion of two modalities may induce gradient competition, leaving both spatial/temporal branches undertrained and leading to mild underfitting). **FPBW-IDS (ours):** Training accuracy is stable at ≈98.0–98.2%; validation accuracy is about 98.0–98.5% with small variance overall and no signs of systematic overfitting.

In summary, across the horizontal comparison, our method (FPBW-IDS) yields consistent gains in validation accuracy over the three baselines: vs. CNN, an improvement of about +0.8 to +1.3 percentage points with lower variance; vs. LSTM, about +0.3 to +0.8 points with smoother convergence; and vs. early-fusion CNN+LSTM, about +1.0 to +1.8 points, while avoiding its tendency toward underfitting and larger fluctuations. Overall, FPBW-IDS converges faster, exhibits fewer and shallower dips, and reaches a higher plateau—demonstrating stronger generalization from the complementary strengths of spatial (Pyramid-DSC), temporal (BiGRU), and frequency (Wavelet) features.

As shown in Fig 12, the training-loss trajectories of the four models differ markedly over epochs: CNN’s training loss decreases slowly to about 0.06, while its validation loss fluctuates considerably (repeatedly spiking to 0.10–0.17), indicating some instability. LSTM converges rapidly early on to around 0.07–0.08; its validation loss is mostly lower than the training loss but with occasional spikes, suggesting that temporal modeling is effective yet sensitive to batched sequence composition. CNN+LSTM (early fusion) exhibits training loss that remains higher than validation loss for a long period (≈0.12–0.16, with occasional extreme peaks), reflecting underfitting and optimization coupling issues.

**Fig 12:**
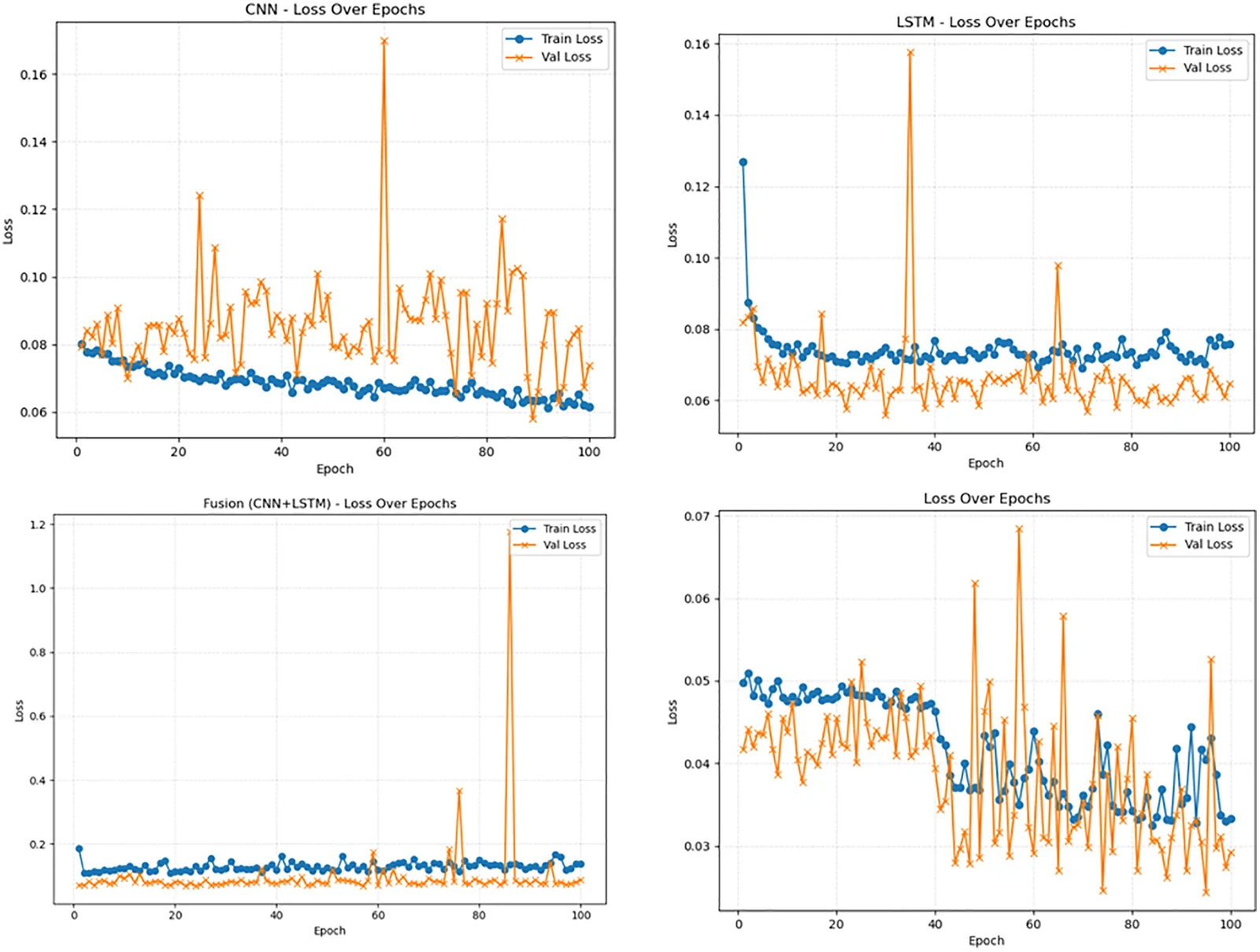
Training and validation loss over epochs: CNN, LSTM, fusion, and proposed model.

In contrast, our method reduces the training/validation losses steadily to about 0.033 / 0.03–0.04, and after 40–60 epochs maintains the smallest generalization gap and the lowest variance, demonstrating better convergence and robustness. Overall, the three-domain fusion strategy—pyramidal depthwise separable convolution (spatial) + BiGRU (temporal) + wavelet (frequency)—is easier to optimize and generalizes better than single-domain baselines and simple early fusion.

The Table 3 presents a comparison of accuracy, precision, recall, and F-score across several deep learning–based intrusion detection models.

**Table 3:** Comparison of accuracy, precision, recall, and F1-score among deep learning–based intrusion detection models.

	Accuracy	Precision	Recall	F-Score
CNN	97.71	87.64	95.16	90.99
LSTM	96.65	91.22	92.53	92.28
CNN-LSTM	96.85	87.44	77.26	78.82
FPBW-IDS	99.39	98.22	93.69	95.56

In summary, among the six models, FPBW-IDS is overall the best (Accuracy 99.39%, F1 95.56%, Precision 98.22%, Recall 93.69%), achieving the best balance between high accuracy and detection rate. DFC-NID attains the second-best F1 (95.44%) with the highest Precision (99.83%), though its recall is slightly lower. CNN is the most sensitive (Recall 95.16%) but its lower Precision (87.64%) leads to an F1 of 90.99%. LSTM performs in the middle of the pack. CNN-LSTM early fusion performs poorly (Recall 77.26%, F1 78.82%). FedSam-AE has the lowest scores across all metrics.

b. After sampling

A comparison of the four confusion matrices shows that, regardless of the approach, the major categories—DoS, DDoS, PortScan, and Patator—maintain near-perfect recall. The differences are mainly concentrated in Normal and the small-sample classes Web Attack and Bot. Among CNN, LSTM, and CNN-LSTM, there remains considerable confusion between Normal and (PortScan/DoS/Bot), and the small-sample classes are quite sensitive to training strategies; of these, CNN-LSTM exhibits the most pronounced spillover. Our method delivers the best overall performance: it significantly reduces misclassification of Normal while clearly improving recall for Web Attack and Bot, achieving more balanced accuracy across categories (Fig 13).

**Fig 13:**
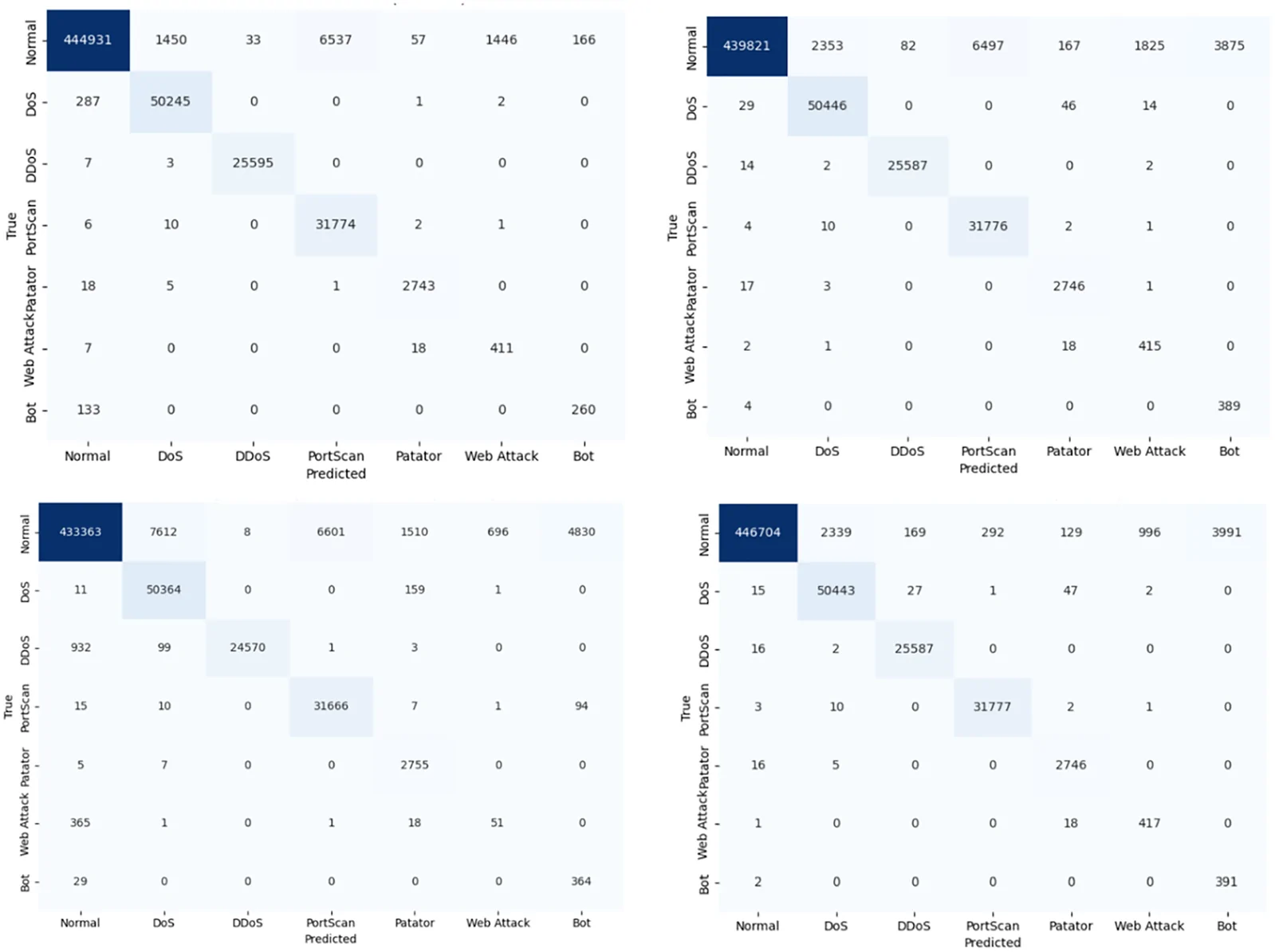
Comparison of accuracy over epochs among CNN, LSTM, fusion, and ours.

As shown in Table 4, the proposed FPBW‑IDS achieves the best overall performance on the evaluation set: Accuracy of 98.99 ± 0.0021%, Recall of 99.29 ± 0.0105%, and F‑Score of 96.89 ± 0.0068%, demonstrating strong robustness and an extremely low miss rate. Its Precision reaches 94.93 ± 0.0227%, which outperforms most compared baselines.

**Table 4:** Comparison of accuracy, precision, recall, and F1-score among deep learning–based intrusion detection models.

	Accuracy(%)	Precision(%)	Recall(%)	F-Score(%)
CNN	97.56 ± 0.0033	92.63 ± 0.0365	93.87 ± 0.0216	91.95 ± 0.0130
LSTM	97.68 ± 0.0054	90.43 ± 0.0381	91.27 ± 0.0078	93.82 ± 0.0313
CNN-LSTM	97.71 ± 0.0046	84.20 ± 0.0212	97.52 ± 0.0140	91.28 ± 0.0071
FPBW-IDS	98.99 ± 0.0021	87.93 ± 0.0227	99.29 ± 0.0105	96.89 ± 0.0068
[28]	95.1	95.0	94.7	94.8
[45]	96.9	96.1	95.5	95.8

The CNN model yields competitive overall results with Accuracy of 97.56 ± 0.0033% and Precision of 92.63 ± 0.0365%, while its Recall is limited to 93.87 ± 0.0216%, corresponding to a relatively conservative decision boundary. The LSTM model obtains relatively balanced yet moderate performance, with an Accuracy of 97.68 ± 0.0054%. The CNN‑LSTM fusion method achieves high Recall at 97.52 ± 0.0140%, yet suffers from degraded Precision of 84.20 ± 0.0212%.

Overall, for intrusion‑detection scenarios where reducing missed attack samples is the top priority, FPBW‑IDS provides the optimal operating point among all compared approaches.

It is worth noting that under the oversampled balanced setting, the model achieves a high recall for minority attack classes at the cost of a moderate drop in precision. As shown in Table 4, the precision decreases to 87.93 ± 0.0227, which corresponds to an increased number of false‑positive predictions. This is a typical precision‑recall trade‑off caused by data‑level oversampling: FCM‑SMOTE densifies minority‑class boundary samples to reduce missed attacks, while inevitably introducing partial classification ambiguity near inter‑class boundaries. For practical high‑stakes IIoT deployment, excessive false positives will produce frequent security alerts and bring heavy workloads to operation‑and‑maintenance personnel. Therefore, further model‑level optimization including cost‑sensitive loss design and classification‑threshold tuning based on precision‑recall curves should be considered to balance false‑alarm rate and attack‑detection performance, which we leave as important future research directions.

Although conventional overall accuracy is prone to majority‑class bias under imbalanced network‑attack distribution, we adopt macro‑F1 and per‑class recall across seven attack categories to reflect the unbiased performance of our approach. Macro‑F1 gives equal weight to each traffic class regardless of sample size, which avoids performance being dominated by majority normal samples. Per‑class recall further reveals the detection capability for rare minority attack classes such as Web Attack and Bot. These metrics can reflect the balancing property of the proposed resampling‑aided detection model. Further dedicated fairness‑aware metrics will be explored in our future work. In addition, all experimental results in this study are obtained with fixed random seeds to ensure reproducibility. Although statistical significance testing through multiple repeated runs can further exclude the interference of random training fluctuations, conducting exhaustive repeated experiments for all comparative models requires substantial computational resources. Therefore, systematic statistical significance verification is not included in the current work and will be explored in our future research.

### (4) UNSW-NB15

To validate the generalization and applicability of the proposed model, experiments are carried out on the UNSW-NB15 dataset. The dataset contains normal traffic and nine typical categories of network attacks: Analysis, Backdoor, DoS, Exploits, Fuzzers, Generic, Reconnaissance, Shellcode and Worms. Analysis is used for network intelligence collection; Backdoor builds illegal remote access channels; DoS attacks paralyze target services through traffic flooding; Exploits obtain privileges by exploiting system flaws; Fuzzers uncover vulnerabilities via malformed input; Generic attacks decrypt encrypted credentials; Reconnaissance performs port and vulnerability detection; Shellcode payloads generate remote command terminals; Worms are self-replicating malware that propagates automatically across networks.

The Fig 14 illustrates the training and validation accuracy curves of CNN, LSTM, baseline model and FPBW-IDS within 100 epochs. The FPBW-IDS model converges fastest, its validation accuracy remains above 0.95 with a smooth curve and minor fluctuations during training, showing superior stability compared with other baselines. Although the pure CNN achieves competitive validation accuracy, its validation curve oscillates violently, leading to unstable training. The LSTM and baseline models obtain lower validation accuracy and suffer from insufficient fitting on the training set, with a relatively large gap between training and validation accuracy. Overall, the proposed FPBW-IDS achieves higher detection accuracy and better training robustness, obtaining the best generalization performance.

**Fig 14:**
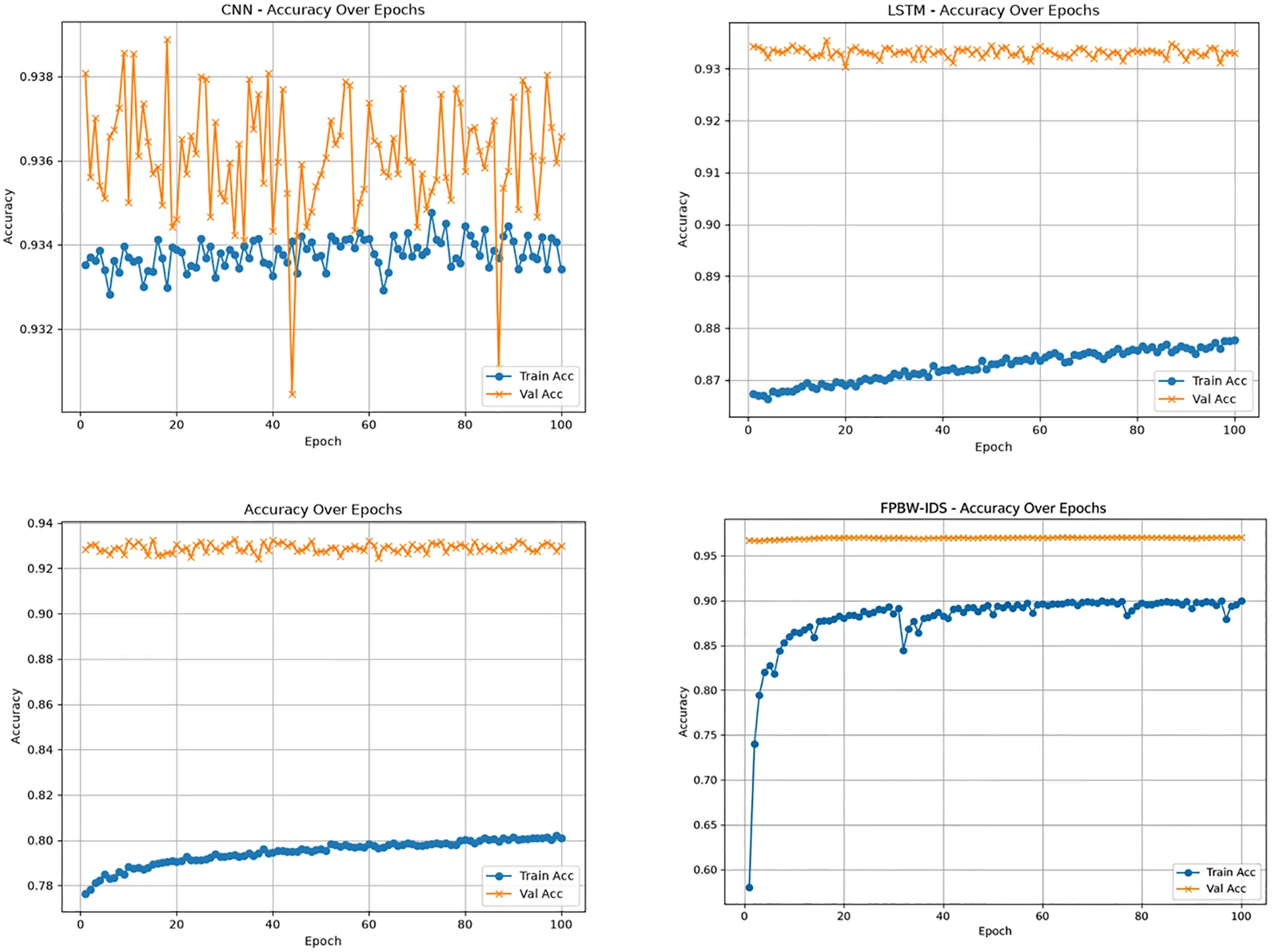
Comparison of accuracy over Epochs among CNN, LSTM, baseline, and ours.

As shown in Fig 15, this paper illustrates the trends of training loss and validation loss of CNN, LSTM, baseline model and FPBW-IDS over 100 training epochs. FPBW-IDS achieves the best loss convergence performance. Its loss drops rapidly at the early stage, and the validation loss decreases synchronously. In the later training stage, the training loss and validation loss are close with smooth curves and a small gap, which indicates no obvious overfitting and stable training. Although the training loss of the pure CNN converges to a low value, its validation loss oscillates violently, leading to large fluctuation of prediction error on the validation set and poor training stability. The training loss of LSTM decreases slowly and remains higher than validation loss with an overall high loss level. The baseline model has the slowest loss reduction and the highest final loss, showing insufficient capability of feature fitting. The comparison demonstrates that FPBW-IDS obtains faster convergence speed, lower steady-state loss and better generalization performance.

**Fig 15:**
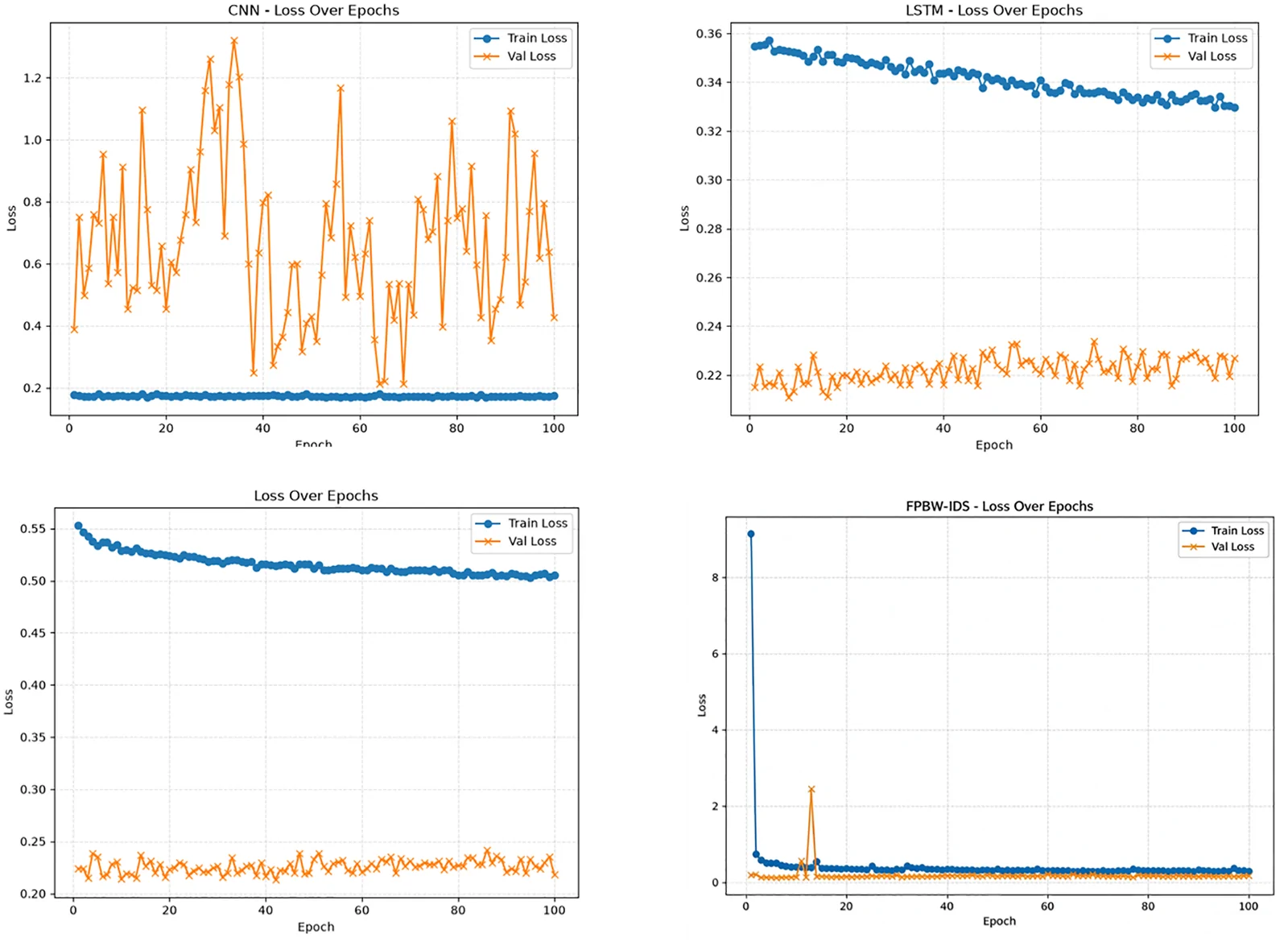
Comparison of loss convergence for CNN, LSTM, baseline and FPBW-IDS.

Fig 16 presents the comparison of Accuracy, Macro Precision, Macro Recall and Macro F1 of FPBW-IDS and other baseline models on the 10% subset of the UNSW-NB15 dataset. The proposed FPBW-IDS achieves the best performance across all four evaluation metrics, surpassing CNN, LSTM, CNN+LSTM, BiGRU, Wavelet and Pyramid DSConv.

**Fig 16:**
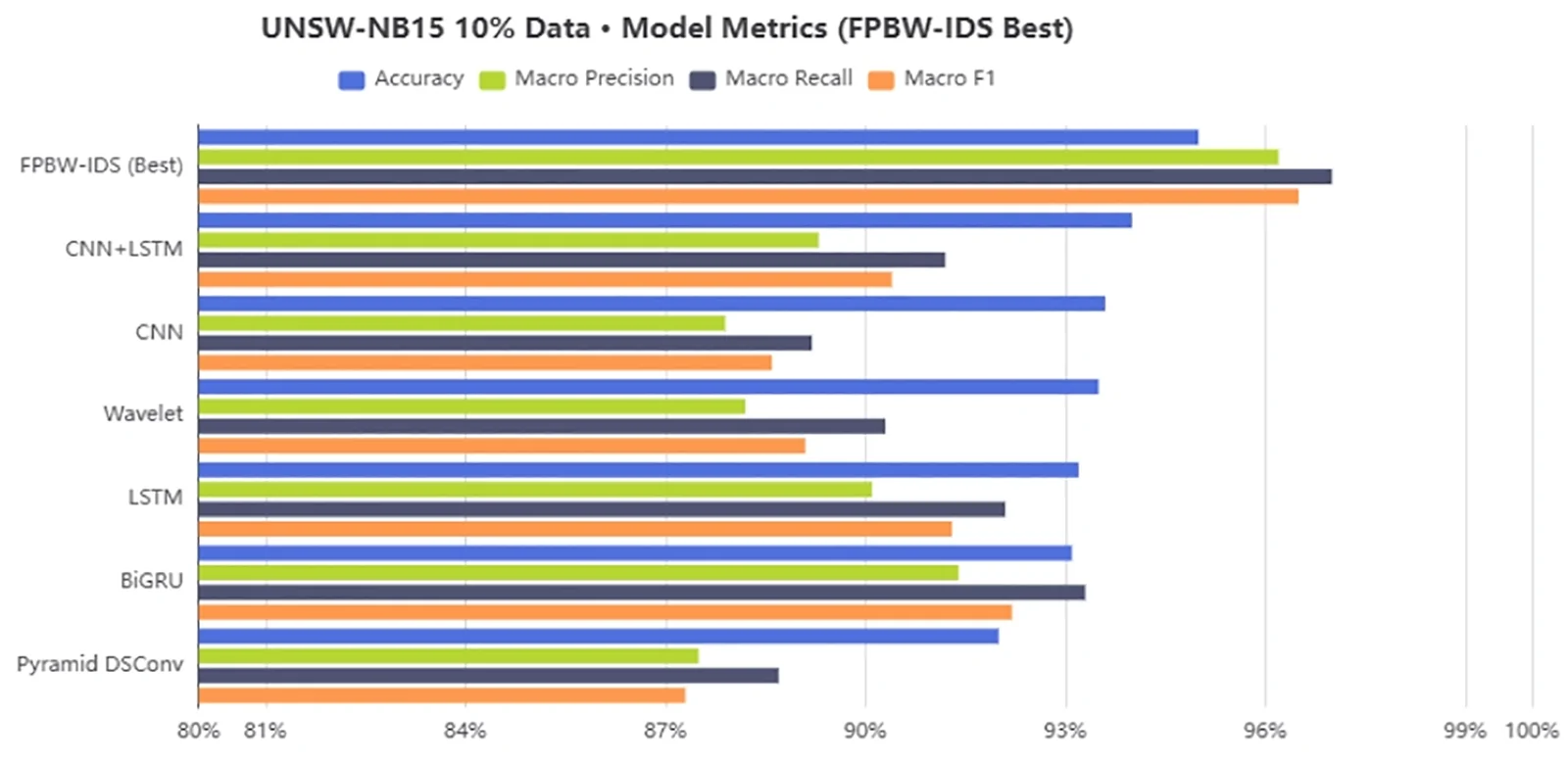
Comparison of evaluation metrics for different models on 10% subset of UNSW-NB15 dataset.

Table 5 presents the comparison results of Accuracy, Macro Precision, Macro Recall and Macro F1 between FPBW-IDS and six baseline models on the UNSW-NB15 dataset. It can be seen that the proposed FPBW-IDS achieves the optimal values across all four evaluation metrics, reaching 95.63%, 96.20%, 97.02% and 96.53%, respectively. Compared with the second-best model, FPBW-IDS improves the Accuracy by 1.52 percentage points over CNN+LSTM. For the macro-averaged metrics, which are more suitable for measuring the recognition performance of minority-class attacks under class-imbalanced scenarios, FPBW-IDS outperforms the second-ranked BiGRU by 4.79, 3.70 and 4.31 percentage points in Macro Precision, Macro Recall and Macro F1, respectively. When compared with the worst-performing Pyramid DSConv model, the Macro F1 of FPBW-IDS increases by as much as 9.23 percentage points, showing remarkable performance superiority.

**Table 5:** Performance comparison of different models on the UNSW-NB15 dataset.

Model	Accuracy(%)	Macro Precision(%)	Macro Recall(%)	Macro F1(%)
FPBW-IDS (Best)	95.63 ± 0.30	96.20 ± 0.25	97.02 ± 0.20	96.53 ± 0.28
CNN+LSTM	94.11 ± 0.42	89.28 ± 0.55	91.24 ± 0.38	90.40 ± 0.48
CNN	93.57 ± 0.50	87.92 ± 0.62	89.20 ± 0.52	88.63 ± 0.44
Wavelet	93.38 ± 0.56	88.15 ± 0.46	90.27 ± 0.54	89.08 ± 0.58
LSTM	93.23 ± 0.40	90.43 ± 0.47	92.17 ± 0.61	91.34 ± 0.49
BiGRU	93.05 ± 0.63	91.41 ± 0.36	93.32 ± 0.41	92.22 ± 0.57
Pyramid DSConv	92.01 ± 0.64	87.50 ± 0.53	88.70 ± 0.66	87.30 ± 0.45

Horizontal comparison of baseline models shows that CNN and Wavelet achieve similar performance, with Accuracy ranging from 93.3% to 93.6%. Benefiting from temporal modeling capability, LSTM and BiGRU obtain higher Macro Recall and Macro F1 than vanilla CNN, indicating that recurrent structures can better extract temporal dependencies within network traffic. Although the CNN+LSTM fusion model achieves an Accuracy of 94.11%, its Macro Precision, Macro Recall and Macro F1 are all lower than those of standalone LSTM and BiGRU. This demonstrates that simple feature concatenation cannot balance the detection performance for majority and minority samples. Moreover, the standard deviations of all metrics of FPBW-IDS are only between 0.20% and 0.30%, which are the smallest among all compared models. This reveals that the model exhibits slight performance fluctuations after multiple independent training runs and possesses favorable stability and reproducibility. In summary, FPBW-IDS significantly outperforms baseline models in both overall detection accuracy and the recognition capability of minority-class attacks.

### (5) Limitations

It should be noted that the 1D‑DWT decomposition applied on reshaped static tabular features belongs to a heuristic data‑driven feature‑engineering strategy, rather than a physically motivated time‑frequency analysis pipeline. Since individual flow samples in the CICIDS2017 dataset are static statistical vectors without inherent temporal order, the constructed one‑dimensional computational dimension is only a computational carrier for multi‑scale feature extraction, rather than authentic time sequence of network traffic. Even so, the extracted multi‑scale coefficients can capture implicit inter‑feature statistical patterns and bring consistent performance gains for imbalanced intrusion‑detection tasks. Future work will explore applying DWT on real session‑based genuine time‑series traffic to further enhance frequency‑domain feature quality.

## 5 Conclusion

In summary, the proposed FPBW-IDS effectively mitigates data imbalance and boundary sparsity through “cluster-prioritized FCM-SMOTE,” while keeping the majority class unchanged (multiplier = 1.0). It further achieves complementary representations and feature-level fusion via three branches spanning the spatial, temporal, and frequency domains (PDSC, BiGRU, and wavelet). On CICIDS2017, the model outperforms CNN, LSTM, and early-fusion baselines in Accuracy, Recall, and F1, markedly reducing missed detections while keeping training/validation curves stable—demonstrating stronger robustness and generalization. This makes it well-suited for deployment in Industrial IoT security scenarios where the cost of missed detections is high.
